# Whole-genome sequencing reveals novel ethnicity-specific rare variants associated with Alzheimer’s disease

**DOI:** 10.1038/s41380-022-01483-0

**Published:** 2022-03-10

**Authors:** Daichi Shigemizu, Yuya Asanomi, Shintaro Akiyama, Risa Mitsumori, Shumpei Niida, Kouichi Ozaki

**Affiliations:** 1 Medical Genome Center, Research Institute, National Center for Geriatrics and Gerontology, Obu, Aichi 474-8511 Japan; 2 Department of Medical Science Mathematics, Medical Research Institute, Tokyo Medical and Dental University (TMDU), Tokyo, 113-8510 Japan; 3 RIKEN Center for Integrative Medical Sciences, Yokohama, Kanagawa 230-0045 Japan

**Keywords:** Molecular biology, Neuroscience, Genetics

## Abstract

Alzheimer’s disease (AD) is the most common multifactorial neurodegenerative disease among elderly people. Genome-wide association studies (GWAS) have been highly successful in identifying genetic risk factors. However, GWAS investigate common variants, which tend to have small effect sizes, and rare variants with potentially larger phenotypic effects have not been sufficiently investigated. Whole-genome sequencing (WGS) enables us to detect those rare variants. Here, we performed rare-variant association studies by using WGS data from 140 individuals with probable AD and 798 cognitively normal elder controls (CN), as well as single-nucleotide polymorphism genotyping data from an independent large Japanese AD cohort of 1604 AD and 1235 CN subjects. We identified two rare variants as candidates for AD association: a missense variant in *OR51G1* (rs146006146, c.815 G > A, p.R272H) and a stop-gain variant in *MLKL* (rs763812068, c.142 C > T, p.Q48X). Subsequent in vitro functional analysis revealed that the *MLKL* stop-gain variant can contribute to increases not only in abnormal cells that should die by programmed cell death but do not, but also in the ratio of Aβ42 to Aβ40. We further detected AD candidate genes through gene-based association tests of rare variants; a network-based meta-analysis using these candidates identified four functionally important hub genes (*NCOR2*, *PLEC*, *DMD*, and *NEDD4*). Our findings will contribute to the understanding of AD and provide novel insights into its pathogenic mechanisms that can be used in future studies.

## Introduction

Alzheimer’s disease (AD) is the most common multifactorial neurodegenerative disease among elderly people [1, 2]. To date, there are no curative treatments for patients who already have AD, and available treatments are only able to delay the progression of the disease [3]. AD is classified into two types according to the age of onset: early-onset AD (EOAD), diagnosed in people <65 years, and late-onset AD (LOAD), diagnosed in people ≥65 years [4]. EOAD accounts for up to 5% of all AD cases, most of which are caused by rare autosomal dominant mutations in one of three genes: amyloid precursor protein (*APP*), presenilin 1 (*PSEN1*), and presenilin 2 (*PSEN2*) [5]. The majority of AD cases are sporadic LOAD, a heterogeneous disorder with complex interactions between genetic and environmental risk factors, which are influenced by multiple common variants with low effect sizes [6, 7]. Estimates of the genetic heritability of LOAD range between 60% and 80% [8]. The ε4 polymorphism in the protein encoded by the apolipoprotein E (*APOE*) gene, located on chromosome 19, is considered to be the strongest genetic risk factor for LOAD [9]. However, the *APOE ε*4 effect accounts for only 27.3% of the overall heritability [10], and a large proportion of the heritability remains unexplained.

Genome-wide association studies (GWAS) have been highly successful in identifying additional genetic risk factors associated with LOAD [7, 11]. Kunkle et al. identified 25 AD risk loci with genome-wide significance by using genomes from over 90000 individuals, including more than 30000 with LOAD [7]. Jansen et al. identified 29 AD risk loci through genome-wide meta-analysis in a case–control study of clinically diagnosed AD (>600,000 individuals) [11]. These findings can explain a fraction of the missing heritability of LOAD. However, GWAS mostly investigate common variants with a minor allele frequency (MAF) ≥ 0.01, which tend to have small effect sizes. Rare variants (MAF < 0.01), which potentially have larger phenotypic effects, have not been sufficiently investigated in LOAD.

The development of next-generation sequencing technologies [12] enables us to detect rare variants. Whole-exome sequencing (WES) and whole-genome sequencing (WGS) have been used to identify rare causal mutations of Mendelian diseases [13, 14] and driver mutations in tumors [15–17]. Although WGS is still less cost-effective than WES, WGS is able to detect more coding variants than WES owing to the limitations of WES capture methods and the advantages of the broader coverage of WGS [18]. Here, we applied WGS to samples from clinically characterized individuals with probable AD and cognitively normal elder controls (CN). We comprehensively investigated pathogenic rare variants associated with AD with single-nucleotide polymorphism (SNP) genotyping data from a large independent Japanese AD case–control cohort. We identified two candidates for rare AD variants: a missense variant in *OR51G1* and a stop-gain variant in *MLKL*. Subsequent in vitro functional analyses demonstrated that the *MLKL* loss-of-function variant plays a crucial role in the pathogenesis of AD. Furthermore, gene-based association tests of rare variants detected AD candidate genes. A subsequent network-based meta-analysis using the candidates revealed functionally important modules (i.e., hub genes). Our findings contribute to the understanding of AD and provide novel insights into its mechanisms of pathogenesis for further investigation in future studies.

## Results

### WGS of Japanese subjects

A total of 3777 samples from 1744 Japanese individuals with AD and 2033 Japanese CN, all at least 60 years old, were included in this study. We performed WGS on 938 of the samples by using the Illumina HiSeq X Ten and NovaSeq 6000 platforms. On average, 365 million read pairs were obtained from the WGS. Of these, 98.3% were mapped to the human reference genome (GRCh37) and 7.6% were removed as duplicate PCR read pairs (Supplementary Table S1). Variant calling was performed by using Genome Analysis Toolkit (GATK) [19]. A total of 15,361,434 genetic variants (single-nucleotide variants [SNVs] and short insertions and deletions [Indels]) from 933 samples passed stringent quality control (QC) criteria for both genotypes and samples (see Materials and Methods). The QC-passed samples consisted of 139 AD and 794 CN samples. Of the 15,361,434 genetic variants, 130,533 were found in protein-coding regions. The average ages of the individuals from whom the AD and CN samples were obtained was 70.5 years (SD = 8.1 years) and 76.5 years (SD = 4.5 years), respectively, and the female-to-male ratios were 1.44:1 and 1.24:1, respectively.

### Variant-based association study for AD pathogenic genes

**Fig. 1:**
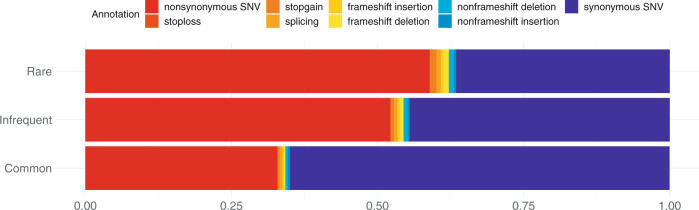
Functional annotations of coding variants. Coding variants were assigned the indicated functional annotations by using ANNOVAR. Of the annotations, frameshift deletion/insertion, splicing variants, stop-gain/stop-loss, and nonsynonymous SNVs were defined as potentially deleterious mutations. On the basis of the minor allele frequency (MAF), available from ToMMo 8.3KJPN, the variants were classified into three groups: rare (MAF < 0.01), infrequent (0.01 ≤ MAF ≤ 0.05), and common (0.05 < MAF).

We classified the 130,533 coding variants into three variant types: rare (*n* = 79,842, 61.2%), infrequent (*n* = 17,100, 13.1%), and common (*n* = 33,591, 25.7%) (see Materials and Methods). We functionally annotated the coding variants. Of the 130,533 variants, 988 were assigned to frameshift deletion; 521 to frameshift insertion: 974 to nonframeshift deletion; 447 to nonframeshift insertion; 856 to splicing variant; 1074 to stop-gain; 76 to stop-loss; 58,611 to synonymous SNV; and 66,986 to nonsynonymous SNV. Rare variants had more potentially deleterious mutations (frameshift deletion/insertion; splicing variants; stop-gain/stop-loss; nonsynonymous SNV) than infrequent and common variants (Fig. 1).

We used functional rare variants for the detection of association signals (discovery data set). The associations were assessed with Fisher’s exact tests. A total of nine genetic markers, located within *FAM126A*, *ZFHX4*, *LGR5, ZFC3H1, OR51G1, OR4X2, ARHGEF4, PRUNE2*, and *MLKL*, were identified as possible associations (*P*_discovery_ < 5.0 × 10^−4^, Table 1). Also, when we examined association signals in all coding variants, one common variant in *APOE*, which has been reported as an AD susceptibility locus in several populations [7, 11], reached the GWAS significance level (rs429358, odds ratio [OR] = 4.23, 95% confidence interval [CI] 2.90–6.15, *P*_discovery_ < 5.0 × 10^−8^, Fig. S1).

**Table 1:** Summary statistics of identified rare coding variants.

Variant	Allele 1/2	Gene	Stage (*n*)	No. of subjects		A1 frequency		*P
				Case	Control	Case	Control	
rs752176038	A/G	*FAM126A*	Discovery (933)	139	794	0.022	0.00063	6.12 × 10^−5^
			Replication (2827)	1596	1231	0.0078	0.0069	1.00
			Combined (3760)	1735	2025	0.0089	0.0044	0.032
rs537717672	T/C	*ZFHX4*	Discovery (927)	137	790	0.026	0.0019	1.09 × 10^−4^
			Replication (2826)	1596	1230	0.0038	0.0061	0.24
			Combined (3753)	1733	2020	0.0055	0.0045	0.62
rs17109924	C/T	*LGR5*	Discovery (932)	138	794	0.025	0.0019	1.10 × 10^−4^
			Replication (2822)	1596	1226	0.0060	0.0045	0.58
			Combined (3754)	1734	2020	0.0075	0.0035	0.024
rs11541286	C/T	*ZFC3H1*	Discovery (932)	138	794	0.025	0.0019	1.10 × 10^−4^
			Replication (2829)	1599	1230	0.0059	0.0045	0.58
			Combined (3761)	1737	2024	0.0075	0.0035	0.024
**rs146006146**	**T/C**	***OR51G1***	**Discovery (933)**	**139**	**794**	**0.032**	**0.0044**	**1.28 × 10**^**−4**^
			**Replication (2798)**	**1585**	**1213**	**0.0091**	**0.0054**	**0.036**
			**Combined (3731)**	**1724**	**2007**	**0.011**	**0.0050**	**1.36 × 10**^**−3**^
rs190260914	A/T	*OR4X2*	Discovery (933)	139	794	0.014	0	4.75 × 10^−4^
			Replication (2822)	1596	1226	0.0038	0.0024	0.48
			Combined (3755)	1735	2020	0.0046	0.0015	0.017
rs1470808145	G/C	*ARHGEF4*	Discovery (933)	139	794	0.014	0	4.75 × 10^−4^
			Replication (2832)	1601	1231	0.0022	0.0028	0.79
			Combined (3765)	1740	2025	0.0032	0.0017	0.24
rs749072077	T/C	*PRUNE2*	Discovery (933)	139	794	0.014	0	4.75 × 10^−4^
			Replication (2829)	1601	1228	0.00062	0.0016	0.41
			Combined (3762)	1740	2022	0.0017	0.00099	0.53
rs55867815	T/G	*MLKL*	Discovery (932)	138	794	0.029	0.0044	4.91 × 10^−4^
			Replication (2827)	1598	1229	0.0084	0.011	0.41
			Combined (3759)	1736	2023	0.010	0.0081	0.39

Bold font indicates statistical significance in both datasets.

*A1* allele 1.

^*^P values were obtained from Fisher’s exact tests.

### Replication study and meta-analysis

For validation assessments, the nine possible association signals identified above were genotyped by using an independent Japanese case–control cohort of 1604 AD and 1235 CN participants (replication data set, Table 1). Of the nine, an *OR51G1* variant (rs146006146, c.815 G > A, p.R272H) showed modest evidence of association in the replication data set (*P*_replication_ < 0.05, Table 1). Subsequent meta-analysis combining results from the discovery and replication datasets showed a significant association with the same direction of effect in both datasets (n = 3731, *P*_meta_ = 1.36 × 10^−3^, Table 1). We further assessed the association by using logistic regression analysis with adjustment for sex and age (*P*_meta_ = 6.82 × 10^−3^, OR = 2.20, 95% CI = 1.24 to 3.91). All of the *OR51G1* variants in WGS were validated by using multiplex PCR-based Invader assay or Sanger sequencing.

The *OR51G1* variant has never been observed in European, American, or African populations except for the population of Asian origin in the Genome Aggregation Database (gnomAD) [20], where the MAF is 0.016 in East Asians. This variant has been observed in other Asian databases, where the MAFs are 0.0049 in the Korean Reference Genome Database (KRGDB) [21] and 0.0089 in the Tohoku Medical Megabank Organization (ToMMo 8.3KJPN) [22], a Japanese database. We have also reported anther olfactory receptor gene (*OR2B2*) from a GWAS analysis of a large number of Japanese AD patients [23]. These results imply that the *OR51G1* variant is an Asian-specific rare AD pathogenic variant.

### Gene-based association study for AD pathogenic genes

**Fig. 2:**
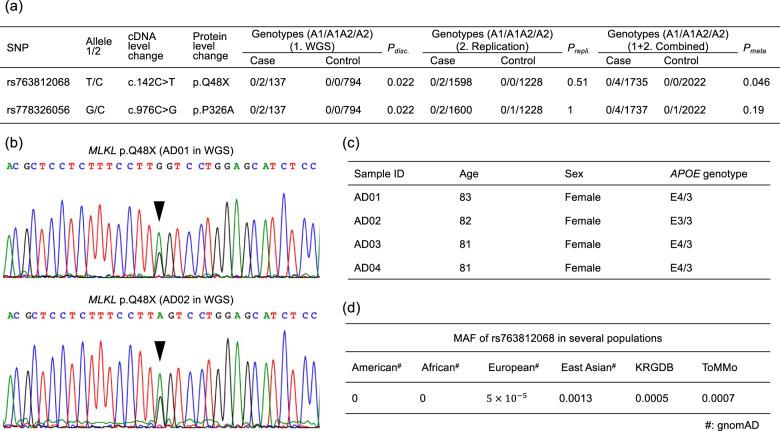
*MLKL* variants identified in a gene-based association study. Two variants (rs763812068 and rs778326056) were genotyped by using a replication data set. **a** For rs763812068, a subsequent meta-analysis combining results from the discovery and replication datasets showed a significant association, with the same direction of effect in both datasets (*P*_meta_ = 0.046). Abbreviations: A1 allele 1, A2 allele 2. **b** *MLKL* stop-gain variants (rs763812068) detected with WGS were validated by using Sanger sequencing. **c** All four of the stop-gain variants were observed in females with AD, most of whom were *APOE ε*4-positive. **d** The stop-gain variant appears especially in Asian populations. The source databases are noted: KRGDB, Korean Reference Genome Database; ToMMo, Tohoku Medical Megabank Organization; gnomAD, Genome Aggregation Database.

In order to identify significant genes with multiple causal variants, we conducted genome-wide gene-based burden testing on rare coding variants. The *MLKL* gene (mixed lineage kinase domain-like) reached a Bonferroni-corrected level of significance (*P*_bon_ = 0.010, Table S2). Six rare variants were involved in the association. Three of them (rs763812068, rs778326056, and rs55867815) showed AD associations with *P* < 0.05 (Table 2). Because rs55867815 had been examined in the variant-based association study and meta-analysis combining results from the discovery and replication datasets showed no significant association (*n* = 3759, *P*_meta_ = 0.39, Table 1), the remaining two (rs763812068 and rs778326056) were further genotyped by using the replication data set. The rs763812068 variant showed a significant association with the same direction of effect in the discovery and replication datasets in a subsequent meta-analysis combining results from both datasets (*P*_meta_ = 0.046, *n* = 3761, Fig. 2a). This is a stop-gain variant (c.142 C > T, p.Q48X) with a high Combined Annotation Dependent Depletion (CADD) Phred-scaled score [24] of 33, defined as a loss-of-function variant, and it was observed only in AD cases (Fig. 2a). All of the stop-gain variants in WGS were validated by using Sanger sequencing (Fig. 2b). All four *MLKL* stop-gain variants were observed in women with AD, of whom three were *APOE ε*4–positive (Fig. 2c). This rare variant appears in Asian populations (0.0013 in gnomAD [20], 0.0005 in KRGDB [21], 0.0007 in ToMMo 8.3KJPN [22]), although it appears rarely in the European population (5.0 × 10^−5^ in gnomAD [20], Fig. 2d).

**Table 2:** Rare variants in the *MLKL* gene associated with Alzheimer’s disease.

Variant	Allele 1/2	cDNA level change	Protein level change	No. of genotypes (A1/A1A2/A2)		A1 frequency		MAF^a^	*P*^b^
				Case	Control	Case	Control		
rs763812068	T/C	c.142 C > T	p.Q48X	0/2/137	0/0/794	0.0072	0	0.0007	**0.022**
rs192549642	G/A	c.288 A > G	p.I96M	0/1/138	0/3/791	0.0036	0.0019	0.0014	0.48
rs1055108157	T/C	c.392 C > T	p.A131V	0/1/138	0/4/790	0.0036	0.0025	0.0018	0.55
rs150170082	A/G	c.865 G > A	p.G289R	0/1/138	0/3/791	0.0036	0.0019	0.0057	0.48
rs778326056	G/C	c.976 C > G	p.P326A	0/2/137	0/0/794	0.0072	0	0.0005	**0.022**
rs55867815	A/C	c.575 C > A	p.S192Y	0/8/130	0/7/787	0.029	0.0044	0.0091	**4.91 × 10**^**−4**^

Bold text indicates statistical significance.

*A1* allele 1, *A2* allele 2, *MAF* Minor allele frequency.

*ToMMo 8.3KJPN* The Tohoku Medical Megabank Organization.

^a^MAFs were obtained from ToMMo 8.3KJPN.

^b^*P* values were obtained from Fisher’s exact tests.

### Functional analysis of the *MLKL* stop-gain variant

**Fig. 3:**
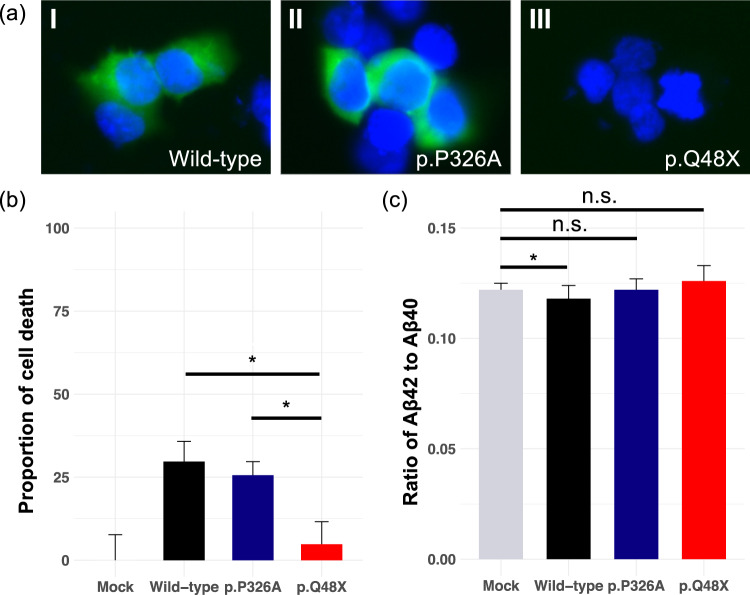
Functional analysis of *MLKL* variants. **a** Localization of Myc-*MLKL* was visualized by immunocytochemistry in human HEK293 cells. Myc: Myc vector. **b** Proportions of cell death using SYTOX Green nuclear stain in human HEK293 cells transfected with the *MLKL* variant proteins. **c** The ratio of Aβ42 to Aβ40 was calculated from the ELISA results. Statistical analysis of the ratio of Aβ42 to Aβ40 between two groups was performed by using Welch’s *t*-test. Statistical significance was set at *P* < 0.05. *: Welch’s *t-*test *P* < 0.05; n.s: not significant.

We conducted in vitro functional analyses for the *MLKL* stop-gain variant (p.Q48X). The cellular localization of variant proteins was first examined by using immunocytochemistry of human HEK293 cells. Wild-type proteins and missense variant proteins (p.P326A) were uniformly distributed around the cell nucleus, whereas we did not observe stop-gain variant proteins in the cells (Fig. 3a). Stop-gain variant proteins were likely degraded to fragments by a nonsense fragment degradation system [25] that acts on abnormal proteins.

*MLKL* is a pseudokinase that functions as the key effector of necroptosis, a form of inflammatory programmed cell death [26]. Therefore, we next assessed the proportion of cell death by using HEK293 cells transfected with the *MLKL* variant proteins. The p.Q48X carrier had a significantly lower proportion of cell death than the wild type (Fig. 3b, Welch’s *t*-test *P* = 0.00030) and p.P326A carrier (Fig. 3b, Welch’s *t*-test *P* = 0.00078). This experiment was independently performed three times with five replicates of each variant carrier (Fig. S2). These results suggest that p.Q48X variant proteins contribute to an accumulation of abnormal cells that should die by programmed cell death but do not because *MLKL* has been inactivated.

Because amyloid-β (Aβ) is implicated in AD pathogenesis [27], we examined whether the *MLKL* variant proteins promote Aβ generation. We transfected the *MLKL* variant proteins into an AD model HEK293 cell line (APPswe-293 cells, see Materials and Methods) and measured the ratio of Aβ42 to Aβ40 by using an enzyme-linked immunosorbent assay (ELISA). The ratio of Aβ42 to Aβ40 was significantly decreased in the wild type compared with the mock-infected cells (Welch’s *t*-test *P* = 0.031), and was increased slightly in the p.Q48X and p.P326A carriers compared with the wild type, whereas it showed no statistically significant difference in p.Q48X and p.P326A carriers compared with the mock-infected cells (Welch’s *t*-test *P* = 0.45, p.Q48X; Welch’s *t*-test *P* = 0.93, p.P326A, Fig. 3c). These results indicated that *MLKL* can play a key role in the decrease of the ratio of Aβ42 to Aβ40. In other words, these findings suggest that the *MLKL* p.Q48X variant can increase the abundance of abnormal cells that would otherwise die by programmed cell death and also contribute to increases in the ratio of Aβ42 to Aβ40.

### Network-based meta-analysis for AD pathogenic genes

**Fig. 4:**
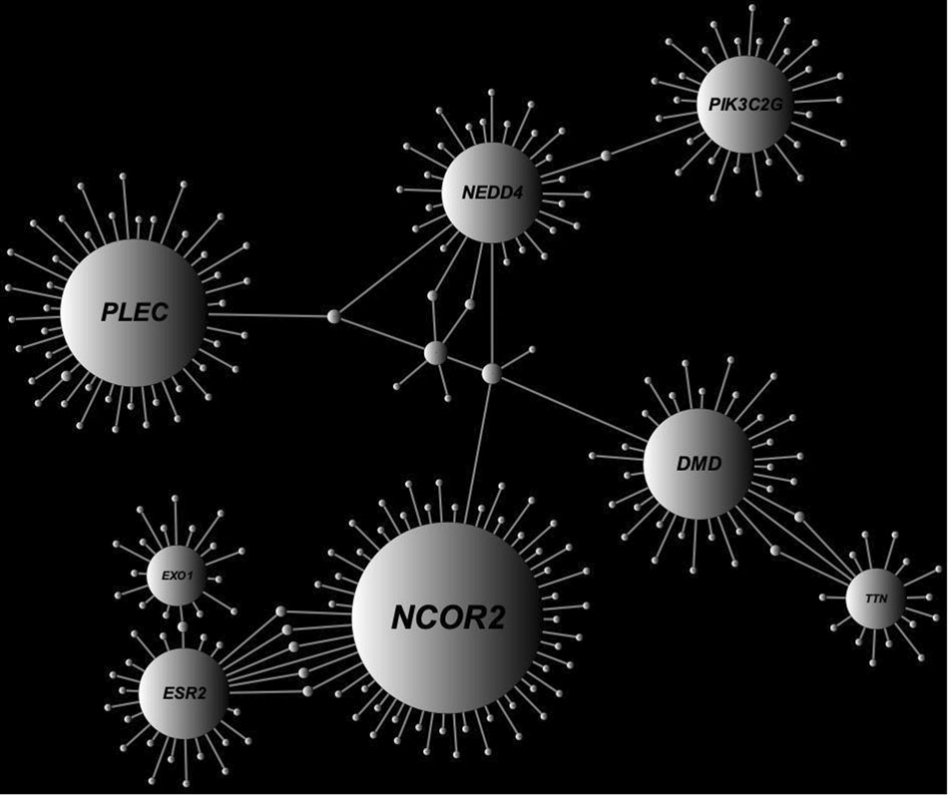
Network-based meta-analysis using candidate pathogenic genes. Through genome-wide gene-based burden testing on rare coding variants, candidate pathogenic genes with *P*_BH_ < 0.1 were detected. A PPI network analysis was performed on the candidates by using NetworkAnalyst with the STRING Interactome database. Eight genes with degree of centrality (DC) ≥15 and betweenness of centrality (BC) ≥3,000 were identified as hub genes. Hub genes size corresponds to the DC. Hub genes are labeled with the gene names.

Protein-protein interaction (PPI) network analysis is effective for identifying functionally important modules (i.e., hub genes) involved in the pathogenesis of AD [28]. Through genome-wide gene-based burden testing on rare coding variants, we detected 67 candidate pathogenic genes with Benjamini-Hochberg (BH) corrected *P*_BH_ < 0.1 (Table S2). We performed a PPI network analysis of the candidate variants by using NetworkAnalyst [29] (http://www.networkanalyst.ca) with the STRING Interactome database [30]. A PPI network with 245 nodes and 254 edges was obtained. The most highly ranked hub genes were recognized in terms of network topology measures of degree of centrality (DC) and betweenness of centrality (BC). Eight top-ranked genes with DC ≥15 and BC ≥3000 were identified as hub genes (*NCOR2*, DC = 56, BC = 17,272; *PLEC*, DC = 43, BC = 9545; *DMD*, DC = 32, BC = 9809.5; *NEDD4*, DC = 29, BC = 14,454; *PIK3C2G*, DC = 28, BC = 6210; *ESR2*, DC = 26, BC = 8177; *EXO1*, DC = 17, BC = 3768; *TTN*, DC = 17, BC = 3311.5, Fig. 4). Four of them have not been verified as expressed in brain tissues through the Human Protein Atlas database [31], which provides quantitative transcriptomics at the tissue and organ level and is publicly accessible at http://www.proteinatlas.org (Fig. S3), but we considered the remaining four (i.e., *NCOR2*, *DMD*, *NEDD4*, and *PLEC*) to be strong candidate hub genes associated with AD pathogenesis.

### Quantitative RT-PCR assay of hub genes by using blood samples

**Fig. 5:**
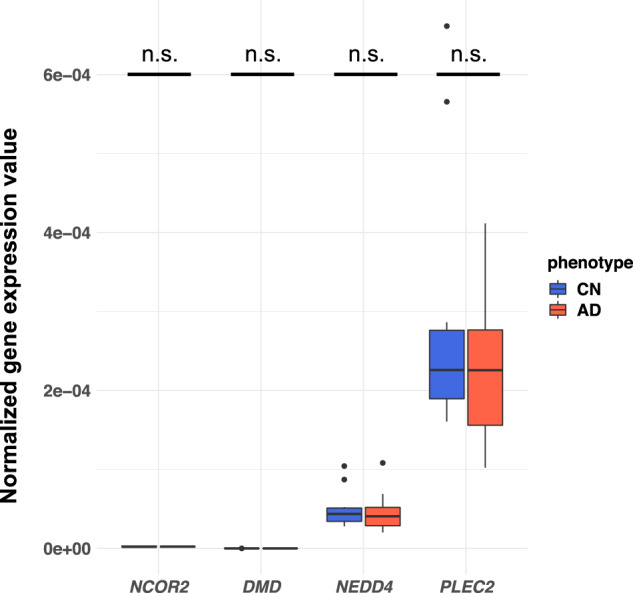
Validation of potential blood biomarkers by using qRT-PCR. The expression of all four hub genes in brain tissues was validated in the Human Protein Atlas database. Differential gene expression between AD and CN subjects (*n* = 20; 10 AD and 10 CN) was investigated. No differences were significant.

For the four hub genes described above (*NCOR2*, *DMD*, *NEDD4*, and *PLEC*), we examined the differential gene expression between AD and CN blood samples, because these genes could have the potential to act as blood-based biomarkers for AD. This would have significant advantages of being more time-and cost-efficient and less invasive than current diagnostic methods for AD. We measured the expression of these genes by using our 20 blood samples (10 ADs and 10 CNs) through quantitative RT-PCR (qRT-PCR). *NCOR2* and *DMD* showed low levels of expression in blood, as shown in the Human Protein Atlas (HPA) database [31] (Fig. S3), so that we could not compare the difference in expression between AD and CN. The remaining two (*PLEC* and *NEDD4*) were expressed in blood, but they showed no statistically significant difference in expression between AD and CN (Welch’s *t*-test *P* = 0.39, *PLEC*; Welch’s *t*-test *P* = 0.77, *NEDD4*, Fig. 5). Unfortunately, these genes cannot be used as blood-based biomarkers for AD prediction, although they might be associated with AD in brain tissues.

## Discussion

By using WGS of 938 subjects and an independent case–control cohort of > 2800 subjects, we identified two novel rare heterozygous variants and four candidate genes in the pathogenesis of AD via variant-based and gene-based association studies and a network-based meta-analysis. A similar analysis was recently conducted on a large number of samples in populations primarily of European descent, and several AD candidate loci have been reported [32]. However, our findings were not included in their results because they seem to be Asian specific.

From our variant-based association study, we first discovered an Asian-specific rare heterozygous missense variant in *OR51G1* showing a nominally significant association with the same direction of effect in the both the discovery and replication sets. *OR51G1* is an olfactory receptor gene, and previous studies have reported a relationship between olfactory dysfunction and risk of cognitive decline [33], including AD [34, 35]. We have also reported another olfactory receptor gene (*OR2B2*) from a GWAS analysis using a large number of Japanese AD patients [23]. These data support a link between our finding and AD pathogenesis.

From our gene-based association study, we discovered a rare heterozygous stop-gain variant, defined as a loss-of-function variant in the *MLKL* gene, which was observed only in samples from individuals with AD among more than 3700 Japanese samples. *MLKL* encodes a pseudokinase that functions as the key effector of necroptosis, a form of inflammatory programmed cell death [26]. We found that the *MLKL* stop-gain variant can decrease the proportion of cells undergoing cell death, presumably leading to the persistence of abnormal cells, and also contribute to increases in the ratio of Aβ42 to Aβ40. In a systematic review, Wang et al. reported that the *MLKL* variant might contribute to late-onset, *APOE* ε4-negative AD in the Chinese population [36]. Faergeman et al. reported from a WGS-based pedigree analysis that a frameshift variant in *MLKL* caused neurodegenerative spectrum disorder; their patients gradually developed mild cognitive impairment [37]. These results strongly support the likelihood that the *MLKL* stop-gain variant contributes to AD pathogenesis, but in contrast to the findings of Wang et al., three of the four carriers identified in this study were *APOE* ε4-positive patients, suggesting that this variant contributes to AD pathogenesis irrespective of *APOE* ε4 allele status in the Japanese population.

From genome-wide gene-based burden testing on rare coding variants, we further detected candidate pathogenic genes. Subsequently, a PPI network analysis based on the candidates elucidated four functionally important hub genes (*NCOR2*, *DMD*, *NEDD4*, and *PLEC*) verified to be expressed in brain tissues through the HPA database [31]. *NCOR2* (nuclear receptor co-repressor 2) binds to several nuclear receptors to suppress the expression of their regulatory targets [38]. The regulatory targets of *NCOR2*, such as *NR4A*, are involved in learning and memory circuits in the adult brain [39]. Zhou et al. reported that loss of function of *NCOR1* and *NCOR2* accompanied memory deficits through a novel GABAergic hypothalamus-CA3 projection [40]. *PLEC* encodes plectin, a universally expressed multifunctional cytolinker protein that is crucial for the intermediate filament network including crosstalk between actomyosin and microtubules [41]. The major isoform, P1c, is expressed in neural and epidermal cells [42]. Valencia et al. reported that lack of P1c in neurons induced long-term memory impairment [41]. The *DMD* (dystrophin) gene is expressed in brain tissues. Anand et al. reported that dystrophin deficiency triggers alterations in the function of central cholinergic synapses and their regulation of neuronal metabolism, and induces cognitive deficits [43]. *NEDD4* encodes the NEDD4 E3 ubiquitin protein ligase, which ubiquitinates the primary mediator of synaptic transmission, AMPA receptors (AMPARs), which are crucial for synaptic plasticity and brain functions [44]. Zhang et al. reported that AMPAR ubiquitination acts as the key molecular event leading to the loss of AMPARs and suppresses synaptic transmission in AD [45]. These data show that all four hub genes can at least be linked to memory impairment.

The two hub genes we were able to evaluate in blood samples (*NEDD4* and *PLEC*) showed no statistical difference in expression in the blood between AD and CN. Therefore, these genes cannot be used as blood-based biomarkers for AD prediction. However, as the expression of all four hub genes may show a statistical difference between AD and CN in brain tissues, cell lines, and animal models, we will continue to explore their potential involvement in the pathogenesis of AD. Although to our knowledge this study is the largest WGS-based rare-variant analysis in Japanese AD cases, further investigation using larger sample sizes will likely reveal additional pathogenic mutations or genes in AD.

In summary, we highlighted two novel ethnicity-specific rare variants (rs146006146, c.815 G > A, p.R272H in *OR51G1*; rs763812068, c.142 C > T, p.Q48X in *MLKL*) and four hub genes (*NCOR2*, *DMD*, *NEDD4*, and *PLEC*) associated with AD pathogenesis. Our findings in this study will not only contribute to the understanding of AD but will also provide insights for future studies into its pathogenic mechanism. In addition, understanding the differences in genetic variant profiles among ethnicities will be important for individualizing treatment and for understanding the pathogenic mechanisms of AD. Association studies between AD and CN using WGS data with a larger sample size would likely lead to the identification of additional novel AD mutations in the future.

## Materials and methods

### Clinical samples

All 3777 blood samples used in this study and the associated clinical data were obtained from the NCGG Biobank. Of the samples, 938 were used for WGS analyses: 140 samples were from patients with AD and 798 samples were from CN. Donors with AD were diagnosed with probable or possible AD by using the criteria of the National Institute on Aging Alzheimer’s Association workgroups [46, 47]. The CN subjects had subjective cognitive complaints but normal cognition on a neuropsychological assessment with a comprehensive neuropsychological test and a Mini-Mental State Examination score >23. Diagnosis of all subjects was based on medical history, physical examination and diagnostic tests, neurological examination, neuropsychological tests, and brain imaging with magnetic resonance imaging or computerized tomography by experts, including neurologists, psychiatrists, geriatricians, or neurosurgeons, all experts in dementia who were familiar with its diagnostic criteria. The remaining 1604 AD subjects and 1235 CN subjects were used as an independent replication cohort for validation assessments. All of the subjects were ≥60 years old.

### WGS data analysis

All WGS data were downloaded from the NCGG Biobank database. DNA concentration was measured by using PicoGreen DNA assay, and fragmentation of DNA was assessed with agarose gel electrophoresis. High-quality DNA was used for DNA libraries. A WGS library was constructed by using the TruSeq DNA PCR-Free Library Preparation Kit (Illumina, Inc., San Diego, CA, USA) in accordance with the manufacturer’s instructions. WGS was conducted at Macrogen Japan Corporation, Takara Bio Inc., and GENEWIZ Inc. DNA was sequenced by using the Illumina HiSeq X Ten or NovaSeq 6000 platform with paired-end reads of 151 bp in accordance with the manufacturer’s instructions.

Read sequences were mapped with BWA-MEM (version 0.7.15) [48] to the human reference genome (GRCh37). Duplicate PCR reads were identified and removed by using picard (version 2.21.4) [49]. Variant calling was conducted by using GATK (version 4.1.0.0) [19]. Individual variant calling was performed by using GATK HaplotypeCaller. Multisample individual variants were joint-called together with in-house data (WGS = 1572) by using GATK GenotypeGVCFs. Variant quality score recalibration was applied according to the GATK Best Practice recommendations [50]. We filtered out SNVs that satisfied the following criteria: (1) Depth (DP) < 10, (2) GenotypeQuality (GQ) < 20, (3) Quality by Depth (QD) < 2, QUAL < 30, StrandOddsRatio (SOR) > 4, FisherStrand (FS) > 60, RMSMappingQuality (MQ) < 40, MappingQualityRankSumTest (MQRankSum) < −12.5, ReadPosRankSumTest (ReadPosRankSum) <−8, and (4) ExcessHet >20, and short Indels with (1) DP < 10, (2) GQ < 20, (3) QD < 2, QUAL < 30, FS > 200, SOR > 10, ReadPosRankSum < −20, and (4) ExcessHet > 20.

QC of the all variants was performed by using PLINK software [51]. We first applied QC filters to the subjects: (1) sex inconsistencies (--check-sex), (2) PI_HAT > 0.25, where PI_HAT is a statistic for the proportion of identity by descent (--genome), (3) genotype missingness (--mind 0.05), and (4) exclusion of outliers from the clusters of East Asian populations in a principal component analysis that was conducted together with population data from 1000 Genomes Phase 3. We next applied QC filters to the variants: (1) genotyping efficiency or call rate (--geno 0.05), (2) minor count (--mac 2), and (3) Hardy-Weinberg equilibrium (--hwe 1 × 10^−5^). Furthermore, we excluded all variants located in low-complexity regions, available from ftp://ftp.1000genomes.ebi.ac.uk/vol1/ftp/release/20130502/supporting/low_-complexity_regions/hs37d5-LCRs.20140224.bed.gz.

### Annotation of variants and variant-based or gene-based association tests

Functional annotations of the variants were made with ANNOVAR (version 20191024). We annotated protein-coding variants with frameshift Indel, nonframeshift Indel, stop-gain/-loss, nonsynonymous SNV, synonymous SNV, and splicing variants. Variant frequency data were found in a public database from 8380 healthy Japanese individuals, called ToMMo 8.3KJPN [22]. We classified the variants into three groups on the basis of MAF: rare (MAF < 0.01), infrequent (0.01 ≤ MAF ≤ 0.05), and common (0.05 < MAF). GWAS of those variants were conducted with Fisher’s exact test by using PLINK software (--model fisher) [51].

Candidate pathogenic variants that satisfied *P* < 5.0 × 10^−4^ were genotyped by using an independent replication cohort. SNP genotyping was performed with the multiplex PCR-based Invader assay (Third Wave Technologies, Madison, WI). Association analysis in the replication cohort as well as the combined analysis of the WGS and replication study were conducted with Fisher’s exact test by using PLINK software (--model fisher) [51]. Variant frequency data were found in public databases: gnomAD [20], KRGDB [21], and ToMMo 8.3KJPN [22].

Gene-based association tests of rare variants-burden tests that assume all variants in the target region have effects on the phenotype in the same direction [52], were implemented in the R programming language (R Development Core Team, http://www.r-project.org/). Genes with Bonferroni-corrected *P*_bon_ < 0.05 were considered to be significantly enriched.

### Construction of plasmids and stable cell lines

Complementary DNA (cDNA) was synthesized from mRNA extracted from the buffy coat of patients. Wild-type *MLKL* was amplified by PCR from the cDNA cloned into a pCMV-Myc vector. The sequence of *MLKL* was confirmed by Sanger sequencing. Variant-type Myc-*MLKL* plasmids, p.Q48X and p.P326A, were constructed from the wild-type Myc-*MLKL* plasmid by using site-directed mutagenesis with PrimeSTAR Max DNA Polymerase (Takara Bio Inc, Shiga, Japan). Primers with each mutation (underlined) were designed in accordance with the manufacturer’s instructions: 5′-CCAGGACTAAGGAAAGAGGAGCGTGC-3′ and 5′-TTTCCTTAGTCCTGGAGCATCTCCAG-3′ for p.Q48X; and 5′-AGAAGCAGCTGAACTCCACGGAAAAA-3′ and 5′-AGTTCAGCTGCTTCTGAATGGTGTAG-3′ for p.P326A. The mutagenesis reaction mix contained 1× PrimeSTAR Max Premix, 0.2 μM of each primer set, and wild-type Myc-*MLKL* plasmid (70 pg) in a total reaction volume of 50 μL. The cycling conditions were 30 cycles of 98 °C for 10 s, 55 °C for 15 s, and 72 °C for 25 s. *Escherichia coli* strain DH5α was transformed with the resulting solutions, and the variant sequences were confirmed by using Sanger sequencing.

Human *APP695* was cloned from human brain cDNA (Clontech, Palo Alto, CA) into a pcDNA™3.1/Hygro (+) vector (Thermo Fisher Scientific, Waltham, MA). Then, the Swedish mutation (APPswe) was induced by using site-directed mutagenesis as described above with the primer set 5′-AAGTGAATCTGGATGCAGAATTCCGAC-3′ and 5′-CATCCAGATTCACTTCAGAGATCTCC-3′. HEK293 cells were transfected with the APPswe plasmid, and cells stably expressing the Swedish mutant of APP695 (HEK-APPswe) were selected in medium containing hygromycin B (Nacalai Tesque, Kyoto, Japan) for 1 week.

### Immunocytochemistry

HEK293 cells were seeded at a density of 2.0 × 10^4^ cells/well on BioCoat Poly-D-Lysine 4-well Culture Slides (Corning, NY, USA), cultured for 24 h, and transfected with wild-type, p.Q48X, or p.P326A Myc-*MLKL* plasmids and FuGENE HD Transfection Reagent (Promega, Madison, WI). Twenty-four hours after transfection, the cells were fixed and incubated with Anti-Myc-tag mAb-Alexa Fluor 488 (MBL, Nagoya, Japan) in accordance with the manufacturer’s protocol. The slides were mounted with SlowFade Diamond Antifade Mountant with DAPI (Thermo Fisher Scientific), and fluorescence images were obtained with a BIOREVO BZ-9000 fluorescence microscope (Keyence, Osaka, Japan).

### Cell death

HEK293 cells were plated in 96-well plates (1.5 × 10^4^ cells/well) and cultured for 24 h before transfection with Myc-*MLKL* plasmids and FuGENE HD (Promega). After 24 h of culture, SYTOX Green (Thermo Fisher Scientific) was added at a final concentration of 5 μM, and the fluorescence intensity was measured at an excitation wavelength of 485 nm and an emission wavelength of 520 nm by using an Infinite M200 PRO plate reader (Tecan, Männedorf, Switzerland). Then the maximum fluorescence intensity for the calculation of cell death rate was measured after all cells were lysed by adding Triton X-100 (final 0.1%). This experiment was performed independently three times with five replicates of each variant carrier.

### Aβ ELISA

HEK293-APPswe cells were plated into 6-well culture plates (4.0 × 10^5^ cells/well) and were cultured for 24 h before transfection with Myc-MLKL plasmids and FuGENE HD (Promega). The supernatant was collected 24 h after transfection, and the concentrations of Aβ40 and Aβ42 were analyzed by using a human β-amyloid ELISA kit (Wako, Osaka, Japan). This experiment was performed independently three times with 12 replicates of each variant carrier. The ratio of Aβ42 to Aβ40 was calculated on the basis of the average of the replicates. Statistical analysis of the ratio of Aβ42 to Aβ40 between two groups was performed with Welch’s *t-*test. Statistical significance was set at a *P* value < 0.05.

### Network-based meta-analysis

Significant genes with multiple causal variants were detected by using genome-wide gene-based burden testing on rare coding variants. Network-based analysis was performed on the genes with a Benjamini-Hochberg (BH) corrected *P*_*BH*_ < 0.1 with NetworkAnalyst 3.0 [29] and the STRING Interactome database [30], which provides comprehensive information about interactions between proteins, including prediction and experimental interaction data. The confidence cutoff score was set to 900. The large-scale PPI network was visualized with Cytoscape v3.8.2 (http://www.cytoscape.org/) [53].

### qRT-PCR validation of genes

cDNA was synthesized by using a PrimeScript II 1st Strand cDNA Synthesis Kit (Takara Bio, Shiga, Japan). qRT-PCR analysis was performed by using TB Green Premix Ex Taq II (Takara Bio, Shiga, Japan) and the Quantstudio7 Flex Real-Time PCR System (Thermo Fisher, Waltham, MA). The following commercially available PCR primers (forward and reverse, 5ʹ to 3ʹ) were used for gene expression analysis: *NCOR2* (GACAAGGAGGCAGAGAAGCCT, GTGACCAGGTGGAGCGTAG), *DMD* (GTTGGAAGAACTCATTACCGCTGC, CTGTCCTAAGACCTGCTCAGC), *NEDD4* (CTGAGGAATTAGAGCCTGGCTG, GCACGTTGTGCTTGCAGTTG), and *PLEC* (GAGTTTGAGAGGCTGGAGTGTC, GGTCTGCACGTCGTTGAAGAG). The qRT-PCR conditions were as follows: one cycle of 50 °C for 2 min, 95 °C for 20 s followed by 40 cycles of 95 °C for 1 s, 60 °C for 20 s, and 72 °C for 30 s. The human beta-2-microglobulin (*hB2M*) was preselected as a reference gene for normalization of target gene expression levels. Gene expression levels from qRT-PCR were calculated relative to that of the reference gene *hB2M* by using the semiquantitative method [54]. Gene expression levels were obtained for 10 AD and 10 CN randomly selected samples.

## Supplementary information

Figure S1

Figure S2

Figure S3

Table S1

Table S2

## Data Availability

All datasets used or analyzed are available from the corresponding author on reasonable request.

## Code availability

We used open source program languages R (version 3.4.1) and Ruby (version 2.4.0) to analyze data and create plots. Code is available upon request from the corresponding authors.
