# Supplementary material for: Whole-genome sequencing reveals novel ethnicity-specific rare variants associated with Alzheimer’s disease
Source: Mol Psychiatry. 2022 Mar 10;27(5):2554–62. doi: 10.1038/s41380-022-01483-0 (PMC9135624; doi:10.1038/s41380-022-01483-0)
Supplement: Supplementary file 1 — Figure S1 [file 41380_2022_1483_MOESM1_ESM.pdf]

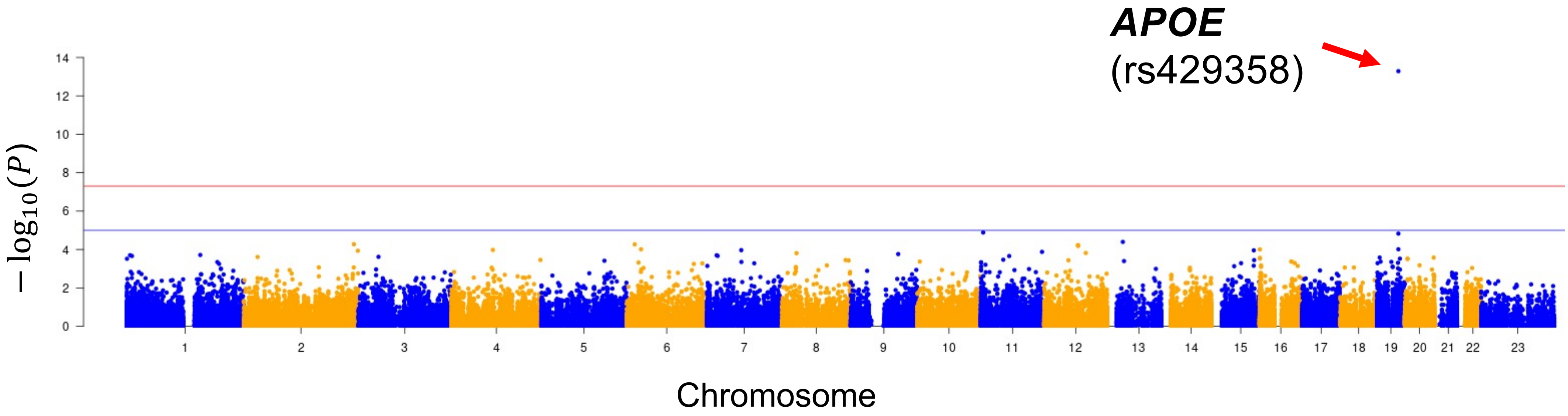

Figure S1. Genome-wide association study of Alzheimer's disease in the Japanese population. The association was assessed with logistic regression analysis adjusted for sex and age.
