# Supplementary material for: Whole-genome sequencing reveals novel ethnicity-specific rare variants associated with Alzheimer’s disease
Source: Mol Psychiatry. 2022 Mar 10;27(5):2554–62. doi: 10.1038/s41380-022-01483-0 (PMC9135624; doi:10.1038/s41380-022-01483-0)
Supplement: Supplementary file 2 — Figure S2 [file 41380_2022_1483_MOESM2_ESM.pdf]

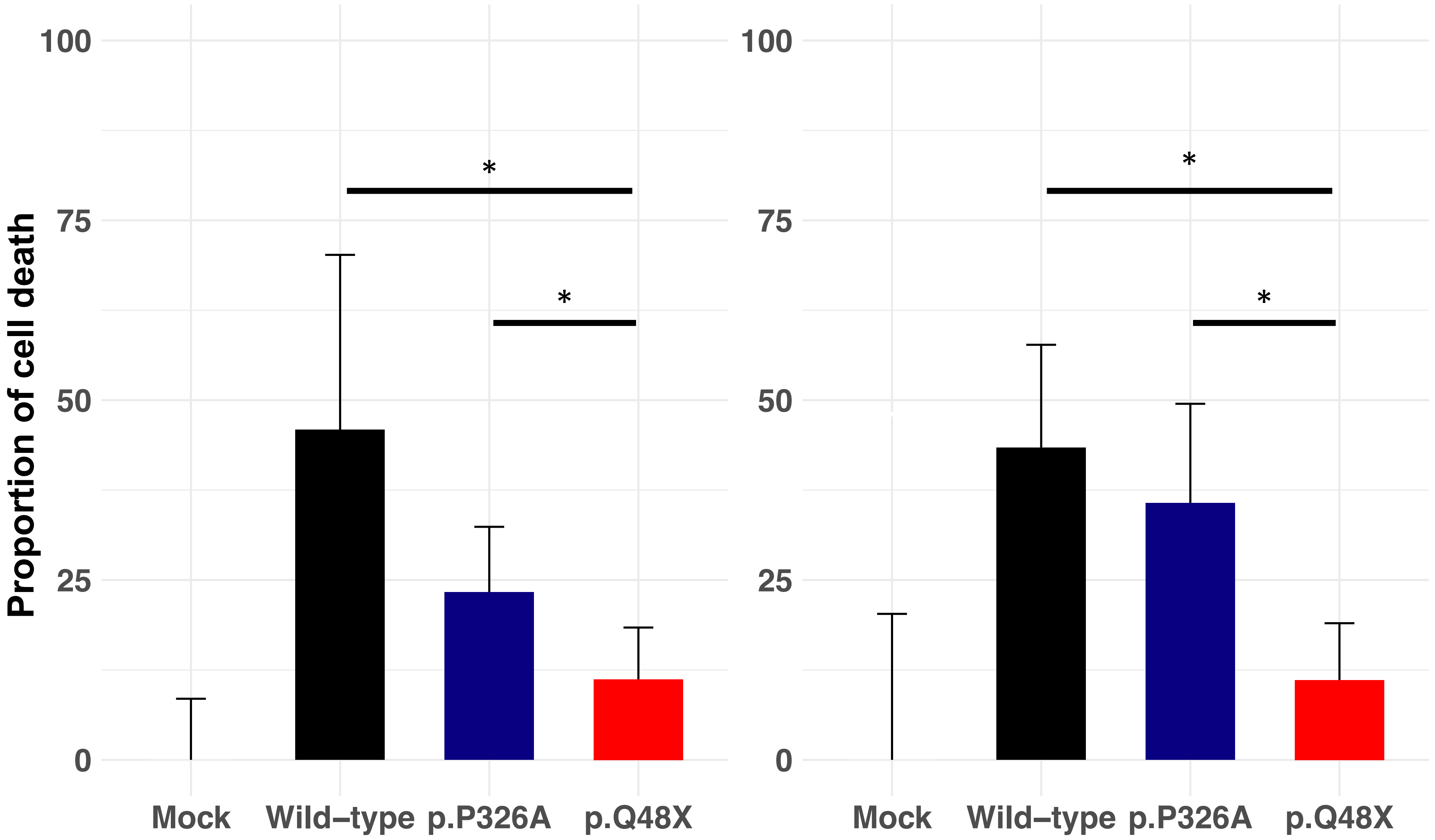

Figure S2. Functional analysis of *MLKL* variants.
Proportions of cell death were determined by using SYTOX Green nuclear stain in human HEK293 cells transfected with the *MLKL* variant proteins. *: Welch’s t-test $P < 0.05$. Each graph represents a separate replication of the experiment.
