# Supplementary material for: Whole-genome sequencing reveals novel ethnicity-specific rare variants associated with Alzheimer’s disease
Source: Mol Psychiatry. 2022 Mar 10;27(5):2554–62. doi: 10.1038/s41380-022-01483-0 (PMC9135624; doi:10.1038/s41380-022-01483-0)
Supplement: Supplementary file 3 — Figure S3 [file 41380_2022_1483_MOESM3_ESM.pdf]

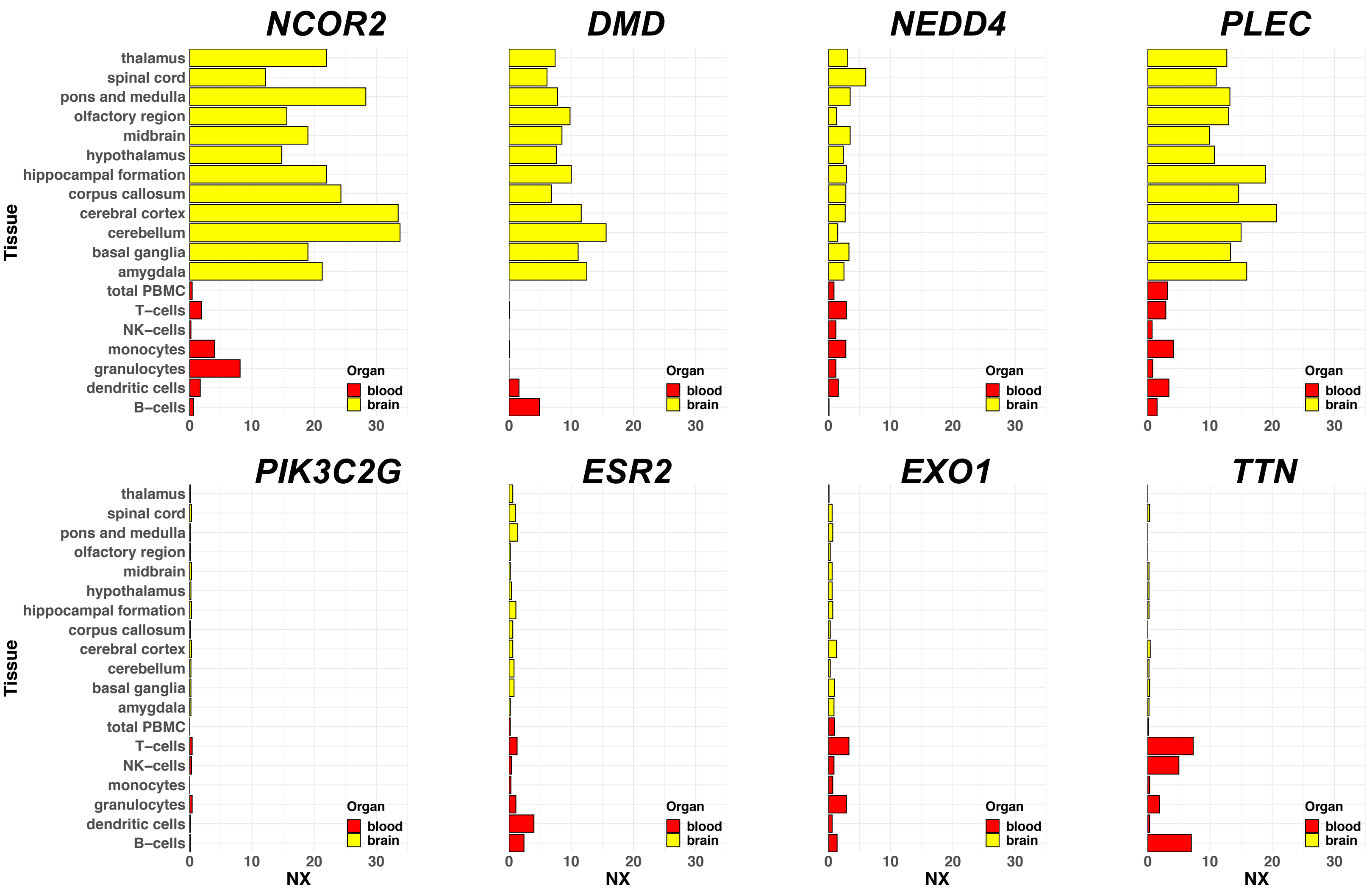

Figure S3. Expression of hub genes detected in the protein-protein interaction network analysis.
The expression of all hub genes in blood cells and brain tissues was checked by using the Human Protein Atlas (HPA) database. An X-axis represents the resulting transcript expression values, denoted normalized expression (NX). PBMDs, peripheral blood monocytes; NK, natural killer.
