# Supplementary material for: Whole-genome sequencing reveals novel ethnicity-specific rare variants associated with Alzheimer’s disease
Source: Mol Psychiatry. 2022 Mar 10;27(5):2554–62. doi: 10.1038/s41380-022-01483-0 (PMC9135624; doi:10.1038/s41380-022-01483-0)
Supplement: Supplementary file 4 — Table S1 [file 41380_2022_1483_MOESM4_ESM.pdf]

Table S1. Overview of 938 WGS performance

Subject ID	Read pairs	Unmapped read pairs	Duplicate PCR read pairs	Mapping rate	PCR duplication rate
AD_1	418346521	5669219	38747288	0.986	0.093
AD_2	428251062	5316662	38175641	0.988	0.089
AD_3	330414277	3529755	28971504	0.989	0.088
AD_4	425351935	5957347	37711020	0.986	0.089
AD_5	332633236	3067351	34396268	0.991	0.103
AD_6	398212016	6511282	35061493	0.984	0.088
AD_7	393749063	7562755	33480020	0.981	0.085
AD_8	309148030	3721811	26249217	0.988	0.085
AD_9	405004141	3544328	36524566	0.991	0.090
AD_10	307138762	6144657	25798122	0.980	0.084
AD_11	397447090	5071712	46557100	0.987	0.117
AD_12	391965968	7212967	33405625	0.982	0.085
AD_13	404607733	6904584	37780208	0.983	0.093
AD_14	421127504	4848169	35771368	0.988	0.085
AD_15	401133544	3649410	41074405	0.991	0.102
AD_16	308694324	2693212	25892907	0.991	0.084
AD_17	418490719	7084699	39094557	0.983	0.093
AD_18	371742135	4787974	33499231	0.987	0.090
AD_19	399190478	6305616	33646488	0.984	0.084
AD_20	320107858	4051562	26154687	0.987	0.082
AD_21	421929473	5453935	57129966	0.987	0.135
AD_22	426339202	6844638	39577507	0.984	0.093

AD_23	310000655	6515686	27380571	0.979	0.088
AD_24	401573958	6583053	38607716	0.984	0.096
AD_25	306842197	7937094	19171273	0.974	0.062
AD_26	398553229	5586676	33308245	0.986	0.084
AD_27	414686861	6724282	39546816	0.984	0.095
AD_28	352210860	3221434	41875953	0.991	0.119
AD_29	414117731	6738863	33662750	0.984	0.081
AD_30	390559787	5190684	32383953	0.987	0.083
AD_31	298647477	2342210	21322155	0.992	0.071
AD_32	425222581	6352832	40234321	0.985	0.095
AD_33	407110679	6407563	39328766	0.984	0.097
AD_34	395504715	6181391	32251526	0.984	0.082
AD_35	338739870	5161994	29704723	0.985	0.088
AD_36	318013658	3328786	26220086	0.990	0.082
AD_37	406295569	6303702	39827237	0.984	0.098
AD_38	398136363	6614396	35089508	0.983	0.088
AD_39	355998400	3028741	39150260	0.991	0.110
AD_40	422016877	4938490	37393923	0.988	0.089
AD_41	320912059	3612459	26625721	0.989	0.083
AD_42	303737735	7340092	20720310	0.976	0.068
AD_43	382307574	5596787	36178033	0.985	0.095
AD_44	422543714	4563574	82361784	0.989	0.195
AD_45	331179179	3165390	28062419	0.990	0.085
AD_46	326399267	4029258	32262641	0.988	0.099

AD_47	346111271	2297571	37012334	0.993	0.107
AD_48	353044598	2594515	40087224	0.993	0.114
AD_49	372112132	2780629	42018543	0.993	0.113
AD_50	370183527	2949524	41616050	0.992	0.112
AD_51	341661005	6500055	23994822	0.981	0.070
AD_52	319134733	8102297	20762016	0.975	0.065
AD_53	398922727	5978926	37239685	0.985	0.093
AD_54	341200556	7332789	27174329	0.979	0.080
AD_55	311545495	8425023	22354834	0.973	0.072
AD_56	371718698	8216908	26452012	0.978	0.071
AD_57	325981781	4185524	41431838	0.987	0.127
AD_58	392469514	6439915	34437769	0.984	0.088
AD_59	406586267	5820273	36603095	0.986	0.090
AD_60	348245941	5058045	37370154	0.985	0.107
AD_61	403111620	3832390	79899300	0.990	0.198
AD_62	576805774	7114201	101143974	0.988	0.175
AD_63	297155601	8682331	19238976	0.971	0.065
AD_64	296516323	3999382	20625726	0.987	0.070
AD_65	414597645	6704496	36725570	0.984	0.089
AD_66	350796063	5107298	39600438	0.985	0.113
AD_67	398265943	6480287	34565738	0.984	0.087
AD_68	454877557	4848882	80283713	0.989	0.176
AD_69	392794130	3844960	46671444	0.990	0.119
AD_70	431652089	2820102	115376228	0.993	0.267

AD_71	296886726	2186960	23184282	0.993	0.078
AD_72	301265563	2914406	24199110	0.990	0.080
AD_73	392961449	5252971	51634616	0.987	0.131
AD_74	307808929	6301545	21176858	0.980	0.069
AD_75	299887991	3077900	24594574	0.990	0.082
AD_76	393045589	6181248	33203583	0.984	0.084
AD_77	296475377	5758957	19526697	0.981	0.066
AD_78	385479767	3448455	32435398	0.991	0.084
AD_79	320253523	7550817	22370134	0.976	0.070
AD_80	369430578	3153809	38654021	0.991	0.105
AD_81	389593003	5309987	59047184	0.986	0.152
AD_82	346950004	2566909	40550201	0.993	0.117
AD_83	352598208	5594780	44095126	0.984	0.125
AD_84	476083189	6079818	72808735	0.987	0.153
AD_85	297292255	7535653	18180793	0.975	0.061
AD_86	396037316	3856094	79023986	0.990	0.200
AD_87	297718235	2976427	21526668	0.990	0.072
AD_88	423336269	9852576	34548495	0.977	0.082
AD_89	385252434	3305321	28765366	0.991	0.075
AD_90	394389564	6543461	33081070	0.983	0.084
AD_91	391823139	4881106	53639558	0.988	0.137
AD_92	298255655	2875543	22272368	0.990	0.075
AD_93	311928451	5543038	23082934	0.982	0.074
AD_94	298593610	2361038	23618347	0.992	0.079

AD_95	304119619	2681464	22425103	0.991	0.074
AD_96	297987907	4385161	20478670	0.985	0.069
AD_97	304057438	3128425	22626743	0.990	0.074
AD_98	394657905	5332762	34032137	0.986	0.086
AD_99	298899192	3559987	21017233	0.988	0.070
AD_100	297144094	2332564	21844391	0.992	0.074
AD_101	416009600	4988459	30737948	0.988	0.074
AD_102	298273833	2556774	22781310	0.991	0.076
AD_103	336077010	7211595	26187116	0.979	0.078
AD_104	441293451	4642828	75790504	0.989	0.172
AD_105	381083369	4371164	28210411	0.989	0.074
AD_106	359543184	3670390	27091469	0.990	0.075
AD_107	349369517	6919713	20411397	0.980	0.058
AD_108	355009502	4906493	51860950	0.986	0.146
AD_109	342706292	4075462	23196920	0.988	0.068
AD_110	345592260	5017358	46163861	0.985	0.134
AD_111	434266799	4112424	65099571	0.991	0.150
AD_112	273004404	2767400	17832923	0.990	0.065
AD_113	347032041	3786753	52511846	0.989	0.151
AD_114	468195352	7035200	63835818	0.985	0.136
AD_115	296286448	4397279	33738661	0.985	0.114
AD_116	306916384	4347942	35482551	0.986	0.116
AD_117	393638987	7640150	34970393	0.981	0.089
AD_118	340242699	6246022	25084166	0.982	0.074

AD_119	420977223	6475413	35240544	0.985	0.084
AD_120	390779889	7907377	33620207	0.980	0.086
AD_121	352781142	3666118	38557529	0.990	0.109
AD_122	307132516	8877848	18933067	0.971	0.062
AD_123	422894495	3899071	83510171	0.991	0.197
AD_124	397722092	7056051	37764333	0.982	0.095
AD_125	328303167	10285810	23866598	0.969	0.073
AD_126	453743028	4631844	85529193	0.990	0.188
AD_127	337614468	8655553	22512294	0.974	0.067
AD_128	356293736	3561610	39225133	0.990	0.110
AD_129	344657290	4883252	37261948	0.986	0.108
AD_130	312933175	7071409	19966972	0.977	0.064
AD_131	357461425	7369824	37664163	0.979	0.105
AD_132	302505362	5095249	23678135	0.983	0.078
AD_133	300171687	8178914	18981847	0.973	0.063
AD_134	295735842	6390561	16099597	0.978	0.054
AD_135	312265972	7947867	21905619	0.975	0.070
AD_136	406163016	9196628	29671959	0.977	0.073
AD_137	401900372	5976110	34691811	0.985	0.086
AD_138	358729067	5311796	36371992	0.985	0.101
AD_139	468276679	7864542	78801608	0.983	0.168
AD_140	331910784	4398422	23963175	0.987	0.072
CN_1	343733195	6167463	24784302	0.982	0.072
CN_2	352224080	4789438	26546978	0.986	0.075

CN_3	370004813	6337634	30999826	0.983	0.084
CN_4	381216709	6770772	30831375	0.982	0.081
CN_5	326832943	4419158	23793973	0.986	0.073
CN_6	351216639	5801979	27748872	0.983	0.079
CN_7	338291256	5495947	26142250	0.984	0.077
CN_8	348330154	6389034	26286489	0.982	0.075
CN_9	359030079	5405679	28888753	0.985	0.080
CN_10	393364431	7167526	29415491	0.982	0.075
CN_11	398628335	7004473	40282188	0.982	0.101
CN_12	361294319	5610711	24790308	0.984	0.069
CN_13	398918418	5703467	35320305	0.986	0.089
CN_14	379141575	7559136	28369575	0.980	0.075
CN_15	403756242	6128872	34372205	0.985	0.085
CN_16	387133804	7758613	28193729	0.980	0.073
CN_17	404072732	6391154	26651644	0.984	0.066
CN_18	393668012	5706181	23600594	0.986	0.060
CN_19	373864878	5259409	23153874	0.986	0.062
CN_20	353024303	6531007	19833104	0.981	0.056
CN_21	373912103	5367705	22680565	0.986	0.061
CN_22	388125859	5489637	24256892	0.986	0.062
CN_23	361359474	7424032	20366406	0.979	0.056
CN_24	398785654	6935847	31451007	0.983	0.079
CN_25	339005420	4987695	18351375	0.985	0.054
CN_26	342520284	6992682	20479139	0.980	0.060

CN_27	379472635	8227136	22802871	0.978	0.060
CN_28	390059009	7012198	25821219	0.982	0.066
CN_29	349543402	5983727	19808837	0.983	0.057
CN_30	391741240	5966038	25210768	0.985	0.064
CN_31	371513563	7648968	19741002	0.979	0.053
CN_32	375766776	6948014	23932768	0.982	0.064
CN_33	371669741	5480942	22179043	0.985	0.060
CN_34	346573964	5235307	20137776	0.985	0.058
CN_35	352392162	7035351	19560888	0.980	0.056
CN_36	357717766	6400294	21798338	0.982	0.061
CN_37	371800359	6703222	19987248	0.982	0.054
CN_38	340549257	6982216	18905947	0.979	0.056
CN_39	346174660	7380388	19022752	0.979	0.055
CN_40	388795153	6111234	25889691	0.984	0.067
CN_41	356647295	6666990	19940925	0.981	0.056
CN_42	326083835	5475781	18491721	0.983	0.057
CN_43	351477201	7804487	20284673	0.978	0.058
CN_44	389154812	5226547	22951861	0.987	0.059
CN_45	344346139	6097995	19419814	0.982	0.056
CN_46	357265193	5715369	19832330	0.984	0.056
CN_47	323633986	6282528	17480754	0.981	0.054
CN_48	380273950	6812684	23744987	0.982	0.062
CN_49	335621777	5105391	18418123	0.985	0.055
CN_50	376801322	6924441	23143985	0.982	0.061

CN_51	347300791	5970355	19969538	0.983	0.057
CN_52	345247639	5681231	19249839	0.984	0.056
CN_53	350070920	6781626	19949881	0.981	0.057
CN_54	370865626	8804534	19814966	0.976	0.053
CN_55	375400151	6534224	22758353	0.983	0.061
CN_56	356488893	6766062	18867895	0.981	0.053
CN_57	337599533	5943666	19013443	0.982	0.056
CN_58	363033714	6984656	20914151	0.981	0.058
CN_59	381161463	7302400	23710704	0.981	0.062
CN_60	369143419	6712571	22190859	0.982	0.060
CN_61	356133777	6973837	20347949	0.980	0.057
CN_62	380155742	9275764	21888740	0.976	0.058
CN_63	347684754	5338241	19608652	0.985	0.056
CN_64	384740106	5967463	28749939	0.984	0.075
CN_65	359445762	5236590	20621613	0.985	0.057
CN_66	372932212	7127398	21880038	0.981	0.059
CN_67	388130744	6722582	24476764	0.983	0.063
CN_68	378067433	5206246	23323420	0.986	0.062
CN_69	377446837	6601366	21642823	0.983	0.057
CN_70	345820553	6502260	18590909	0.981	0.054
CN_71	384542605	6971744	26224667	0.982	0.068
CN_72	356705599	5833898	20173045	0.984	0.057
CN_73	327882880	6179909	21920672	0.981	0.067
CN_74	344270482	5478994	23898076	0.984	0.069

CN_75	373314207	6876939	27063595	0.982	0.072
CN_76	332629114	5875906	21758829	0.982	0.065
CN_77	325133890	5353228	21716111	0.984	0.067
CN_78	374115778	6984823	26177213	0.981	0.070
CN_79	325396659	4945296	21783773	0.985	0.067
CN_80	328390251	5244616	19723230	0.984	0.060
CN_81	371316742	5340856	28233373	0.986	0.076
CN_82	324344237	5247786	20465354	0.984	0.063
CN_83	331613954	4913877	20966315	0.985	0.063
CN_84	357207818	5870481	26821989	0.984	0.075
CN_85	339644662	6581343	22294775	0.981	0.066
CN_86	320193378	6013021	20003218	0.981	0.062
CN_87	338510233	6384001	21456714	0.981	0.063
CN_88	351252826	5500568	22498273	0.984	0.064
CN_89	343995674	6101595	22998715	0.982	0.067
CN_90	351725960	6825770	23246809	0.981	0.066
CN_91	347530707	6375412	22440983	0.982	0.065
CN_92	345314399	6477691	22883727	0.981	0.066
CN_93	367435572	5729656	25551302	0.984	0.070
CN_94	380088377	6463953	26351783	0.983	0.069
CN_95	346707944	5328021	22252827	0.985	0.064
CN_96	364820327	6564452	24610933	0.982	0.067
CN_97	343560752	5603724	22806731	0.984	0.066
CN_98	377330069	7778966	26515323	0.979	0.070

CN_99	384781819	7894532	24377805	0.979	0.063
CN_100	396098269	6175652	34642564	0.984	0.087
CN_101	361258342	6093466	21626183	0.983	0.060
CN_102	391689498	5523318	29841312	0.986	0.076
CN_103	385967520	6970556	25581276	0.982	0.066
CN_104	399460977	6707753	30295112	0.983	0.076
CN_105	378877279	7502901	24851940	0.980	0.066
CN_106	389192442	7200208	29614763	0.981	0.076
CN_107	391718224	7081094	27451684	0.982	0.070
CN_108	369387034	7289062	25896505	0.980	0.070
CN_109	350261773	6524357	22940329	0.981	0.065
CN_110	350068589	7056685	22390793	0.980	0.064
CN_111	366847416	5154102	25840102	0.986	0.070
CN_112	386142619	6658610	27558874	0.983	0.071
CN_113	340025268	6942711	21710988	0.980	0.064
CN_114	376426937	5315080	31135497	0.986	0.083
CN_115	352511203	6245135	23064265	0.982	0.065
CN_116	360676937	5508188	24435073	0.985	0.068
CN_117	405489697	7700682	25629448	0.981	0.063
CN_118	372906980	7217401	24571852	0.981	0.066
CN_119	387252278	6525625	23728209	0.983	0.061
CN_120	384158229	8047480	21414821	0.979	0.056
CN_121	336981298	5860906	17517426	0.983	0.052
CN_122	386537800	7585248	25458550	0.980	0.066

CN_123	344567876	6766372	19101388	0.980	0.055
CN_124	373671210	6637182	21555730	0.982	0.058
CN_125	349109765	7138889	19842842	0.980	0.057
CN_126	387576734	6040651	24301651	0.984	0.063
CN_127	339401361	5304318	19150553	0.984	0.056
CN_128	333909183	6644587	17160230	0.980	0.051
CN_129	389777227	8442438	25582641	0.978	0.066
CN_130	356704651	6983501	19616106	0.980	0.055
CN_131	340672371	6044493	17198042	0.982	0.050
CN_132	386323909	8763928	24482904	0.977	0.063
CN_133	356351306	6078459	19975494	0.983	0.056
CN_134	321448074	5061450	17527454	0.984	0.055
CN_135	342514281	5505059	18504591	0.984	0.054
CN_136	359847949	6887766	20650002	0.981	0.057
CN_137	354163492	7003706	20222500	0.980	0.057
CN_138	362730131	9346831	20707543	0.974	0.057
CN_139	351153523	6670624	19939489	0.981	0.057
CN_140	372373666	5898273	21323794	0.984	0.057
CN_141	391014489	6781233	23188301	0.983	0.059
CN_142	338808135	5249364	18376580	0.985	0.054
CN_143	349820814	6873464	20894551	0.980	0.060
CN_144	357055762	5123798	20883831	0.986	0.058
CN_145	374788956	6913158	23619832	0.982	0.063
CN_146	383882261	7769010	21891789	0.980	0.057

CN_147	391105466	7509729	30385563	0.981	0.078
CN_148	343767084	6574966	18846823	0.981	0.055
CN_149	394496451	7150955	25928402	0.982	0.066
CN_150	383350586	6414254	23487405	0.983	0.061
CN_151	385806868	8355924	23782136	0.978	0.062
CN_152	387983498	7307859	25845597	0.981	0.067
CN_153	388278067	6799623	25410719	0.982	0.065
CN_154	389106466	6842361	22508418	0.982	0.058
CN_155	371082777	7250574	23358357	0.980	0.063
CN_156	370488114	5781832	22670392	0.984	0.061
CN_157	380763078	6361407	23478504	0.983	0.062
CN_158	346959908	8533008	19793958	0.975	0.057
CN_159	391191262	7869401	31517615	0.980	0.081
CN_160	335455001	7057233	17857909	0.979	0.053
CN_161	346877625	7243946	20020914	0.979	0.058
CN_162	366205885	6270911	22071965	0.983	0.060
CN_163	348692240	5385685	19569757	0.985	0.056
CN_164	383758889	6828816	24275326	0.982	0.063
CN_165	360270268	7043598	19003787	0.980	0.053
CN_166	378223659	6755720	23717149	0.982	0.063
CN_167	358168018	4582302	20747331	0.987	0.058
CN_168	370993547	6902801	38647511	0.981	0.104
CN_169	369908578	7653316	35034729	0.979	0.095
CN_170	393850172	9107380	41367476	0.977	0.105

CN_171	375184051	9352685	35648788	0.975	0.095
CN_172	397412363	9188059	37397222	0.977	0.094
CN_173	371765315	7181416	37620040	0.981	0.101
CN_174	393722206	7735331	43572076	0.980	0.111
CN_175	369872343	5923479	35661646	0.984	0.096
CN_176	354887006	5681041	35458589	0.984	0.100
CN_177	356562062	8500386	30795102	0.976	0.086
CN_178	382099355	6638373	43277255	0.983	0.113
CN_179	379540976	6719353	35312430	0.982	0.093
CN_180	378598619	7741808	38334913	0.980	0.101
CN_181	382500889	6405995	37904638	0.983	0.099
CN_182	364253160	6710328	39575085	0.982	0.109
CN_183	376651917	7197215	35466670	0.981	0.094
CN_184	376891082	6870638	35697162	0.982	0.095
CN_185	367738851	6110916	35341220	0.983	0.096
CN_186	380268259	6351517	39721398	0.983	0.104
CN_187	375299208	6581224	37248084	0.982	0.099
CN_188	386447335	7947055	36626188	0.979	0.095
CN_189	380045988	7733640	37162174	0.980	0.098
CN_190	379944552	7662714	35273011	0.980	0.093
CN_191	387699686	7147891	41507631	0.982	0.107
CN_192	374814256	9107075	35788581	0.976	0.095
CN_193	376254457	9254958	36219613	0.975	0.096
CN_194	378025900	7640342	37038275	0.980	0.098

CN_195	377592385	7285254	36628296	0.981	0.097
CN_196	385158154	8048775	35857387	0.979	0.093
CN_197	384128149	8413897	39529233	0.978	0.103
CN_198	377374616	6830554	37019737	0.982	0.098
CN_199	369145641	6912705	44028717	0.981	0.119
CN_200	349886255	7510513	32321059	0.979	0.092
CN_201	362095387	6865597	35715065	0.981	0.099
CN_202	366752423	6005910	37469475	0.984	0.102
CN_203	361200763	7948692	32991224	0.978	0.091
CN_204	343167665	5618623	31948753	0.984	0.093
CN_205	354790354	6157656	32332072	0.983	0.091
CN_206	357439310	6118282	34030516	0.983	0.095
CN_207	354102235	8272535	33781155	0.977	0.095
CN_208	355994658	5583581	35771693	0.984	0.100
CN_209	367418364	7373950	35617539	0.980	0.097
CN_210	360084844	6125343	34663118	0.983	0.096
CN_211	383077978	7470428	38134881	0.980	0.100
CN_212	389718146	9839271	36845299	0.975	0.095
CN_213	379929841	6796924	38145270	0.982	0.100
CN_214	360444243	8072151	36014025	0.978	0.100
CN_215	356690311	9560544	34616101	0.973	0.097
CN_216	339212219	5602690	32523994	0.983	0.096
CN_217	330024589	3702358	34087591	0.989	0.103
CN_218	332776059	5559885	32696655	0.983	0.098

CN_219	340073094	5711104	31447085	0.983	0.092
CN_220	335837221	5943943	31805026	0.982	0.095
CN_221	330399590	4608367	29729254	0.986	0.090
CN_222	359869259	5036730	37521343	0.986	0.104
CN_223	334491921	6481855	29437604	0.981	0.088
CN_224	330479630	5305390	30942742	0.984	0.094
CN_225	331679820	5137568	31624276	0.985	0.095
CN_226	352814987	7564301	31908940	0.979	0.090
CN_227	361063304	5035631	37055579	0.986	0.103
CN_228	334105639	5154348	31737096	0.985	0.095
CN_229	337028141	5598767	30195920	0.983	0.090
CN_230	350718235	7752519	35032575	0.978	0.100
CN_231	335831866	5317395	31970756	0.984	0.095
CN_232	388746822	5715917	45430772	0.985	0.117
CN_233	328835645	5910593	30665682	0.982	0.093
CN_234	343166588	5081413	33570768	0.985	0.098
CN_235	385201971	5429341	37332259	0.986	0.097
CN_236	363663174	5846569	38768442	0.984	0.107
CN_237	333001001	4786413	33703319	0.986	0.101
CN_238	336905690	4522451	31216668	0.987	0.093
CN_239	383455464	4879931	43690543	0.987	0.114
CN_240	329718345	5810689	30362216	0.982	0.092
CN_241	330177302	4645002	30838308	0.986	0.093
CN_242	339097416	5434595	33304604	0.984	0.098

CN_243	335972951	4554642	33251317	0.986	0.099
CN_244	344778881	6741508	32685707	0.980	0.095
CN_245	338305895	5099263	32905593	0.985	0.097
CN_246	337490040	4227084	33895251	0.987	0.100
CN_247	341361907	4477008	32491859	0.987	0.095
CN_248	342246193	6669916	29968667	0.981	0.088
CN_249	357133472	7769647	37839018	0.978	0.106
CN_250	366862448	8219781	32243107	0.978	0.088
CN_251	382164793	6915446	40357375	0.982	0.106
CN_252	335915095	5151779	20944051	0.985	0.062
CN_253	331340227	4734599	20825563	0.986	0.063
CN_254	346816437	6796872	21331338	0.980	0.062
CN_255	353293552	6638472	28899017	0.981	0.082
CN_256	363262819	8265524	33881032	0.977	0.093
CN_257	377456855	6850327	34642149	0.982	0.092
CN_258	362915632	6643341	34253096	0.982	0.094
CN_259	374003952	7007333	35183213	0.981	0.094
CN_260	360181111	7089494	33710270	0.980	0.094
CN_261	368425597	9239077	33523198	0.975	0.091
CN_262	371924477	7759528	35447507	0.979	0.095
CN_263	385813198	6564656	38669393	0.983	0.100
CN_264	386240379	7795293	37557493	0.980	0.097
CN_265	370116646	7683179	34912467	0.979	0.094
CN_266	362790853	7977331	32731228	0.978	0.090

CN_267	369593741	8294627	36305751	0.978	0.098
CN_268	361846279	5639641	44014268	0.984	0.122
CN_269	371315218	6609359	32412255	0.982	0.087
CN_270	360835779	5851022	38096787	0.984	0.106
CN_271	361682833	5881589	32785308	0.984	0.091
CN_272	366632826	6135908	37486203	0.983	0.102
CN_273	358549188	7151549	31671255	0.980	0.088
CN_274	368033788	7545578	38456475	0.979	0.104
CN_275	359048834	8992648	32335827	0.975	0.090
CN_276	345600765	6436354	21035892	0.981	0.061
CN_277	330919310	7106641	19018811	0.979	0.057
CN_278	364663820	6048784	22292219	0.983	0.061
CN_279	354606905	6330587	23117561	0.982	0.065
CN_280	322565103	5464711	20582660	0.983	0.064
CN_281	335076780	6108547	20472485	0.982	0.061
CN_282	354723379	5529438	22352428	0.984	0.063
CN_283	334883014	7293892	21236080	0.978	0.063
CN_284	335523044	5154816	21517580	0.985	0.064
CN_285	332603247	6183254	20937242	0.981	0.063
CN_286	358332075	5086338	23275131	0.986	0.065
CN_287	336019536	4813053	21448872	0.986	0.064
CN_288	351677794	5550832	20426472	0.984	0.058
CN_289	328129559	4705724	20981260	0.986	0.064
CN_290	337147111	4484494	20930226	0.987	0.062

CN_291	348311128	5391453	21828849	0.985	0.063
CN_292	336851201	6121109	22082029	0.982	0.066
CN_293	333526013	4607308	19872850	0.986	0.060
CN_294	372551266	6344620	24144400	0.983	0.065
CN_295	331826931	5474399	19498710	0.984	0.059
CN_296	350010223	6272306	24113608	0.982	0.069
CN_297	352256349	5383123	22492488	0.985	0.064
CN_298	333459888	6160706	20439664	0.982	0.061
CN_299	333709798	3546739	20225635	0.989	0.061
CN_300	350515921	5286310	22016244	0.985	0.063
CN_301	349085699	6302331	22947159	0.982	0.066
CN_302	332577305	4996070	19801014	0.985	0.060
CN_303	370348849	5909730	22811571	0.984	0.062
CN_304	322525714	4125818	21745300	0.987	0.067
CN_305	357751609	5301444	22090810	0.985	0.062
CN_306	323100225	4926844	20552127	0.985	0.064
CN_307	345507399	6584961	21553367	0.981	0.062
CN_308	363708830	5660141	23188418	0.984	0.064
CN_309	355065748	5854487	22546622	0.984	0.063
CN_310	348603648	6429301	22046868	0.982	0.063
CN_311	337529797	5513885	22505369	0.984	0.067
CN_312	353339692	6389378	21611012	0.982	0.061
CN_313	331134451	6126113	20199048	0.981	0.061
CN_314	352063215	4877490	22837976	0.986	0.065

CN_315	349137082	5098519	26112905	0.985	0.075
CN_316	334540617	4535856	20578483	0.986	0.062
CN_317	319633273	5567795	19125608	0.983	0.060
CN_318	352728944	5317285	22463414	0.985	0.064
CN_319	332648014	4227828	21743564	0.987	0.065
CN_320	336136965	4707319	21579864	0.986	0.064
CN_321	323833037	5473544	19091357	0.983	0.059
CN_322	353553542	6094191	21717954	0.983	0.061
CN_323	327225932	3958678	22772241	0.988	0.070
CN_324	363072163	7665894	22488983	0.979	0.062
CN_325	378382093	7033881	24793109	0.981	0.066
CN_326	318539720	4264205	18833547	0.987	0.059
CN_327	376059812	8365726	23893288	0.978	0.064
CN_328	348589197	6387606	19291577	0.982	0.055
CN_329	363868229	5541657	24295716	0.985	0.067
CN_330	355570761	4249817	21983329	0.988	0.062
CN_331	330456136	6576194	20002312	0.980	0.061
CN_332	356806152	5995526	22641593	0.983	0.063
CN_333	350074505	5955415	20208020	0.983	0.058
CN_334	342437318	6348526	19532362	0.981	0.057
CN_335	353137761	6606172	19772672	0.981	0.056
CN_336	401390374	7803943	27481778	0.981	0.068
CN_337	357736165	4895432	20828282	0.986	0.058
CN_338	327313421	6655861	19153876	0.980	0.059

CN_339	359653454	7098879	21491671	0.980	0.060
CN_340	384849398	4862415	23394149	0.987	0.061
CN_341	347264628	5947448	20667862	0.983	0.060
CN_342	360158727	7792268	23629476	0.978	0.066
CN_343	372107123	6851147	21019387	0.982	0.056
CN_344	324708809	5321773	19057403	0.984	0.059
CN_345	392113646	6009509	24979629	0.985	0.064
CN_346	326726060	5769785	19097964	0.982	0.058
CN_347	381977201	8429368	25129225	0.978	0.066
CN_348	343972522	5855633	20969413	0.983	0.061
CN_349	343614165	5866269	19885709	0.983	0.058
CN_350	350862936	6707209	20796770	0.981	0.059
CN_351	369373508	6994340	20966634	0.981	0.057
CN_352	380782593	7199864	24925735	0.981	0.065
CN_353	341923581	7127135	19092236	0.979	0.056
CN_354	345375333	6816473	20845038	0.980	0.060
CN_355	371464732	6793906	22449391	0.982	0.060
CN_356	389455705	6912138	25274703	0.982	0.065
CN_357	365200043	5646001	22675090	0.985	0.062
CN_358	342516299	5659537	19856006	0.983	0.058
CN_359	381893608	8640071	23447822	0.977	0.061
CN_360	348484008	6404003	20490898	0.982	0.059
CN_361	389426268	6228124	28435737	0.984	0.073
CN_362	360468465	6042547	21020080	0.983	0.058

CN_363	376494194	7023928	22992300	0.981	0.061
CN_364	385014465	6246187	24328490	0.984	0.063
CN_365	379013462	8066414	25465904	0.979	0.067
CN_366	386691950	6894424	29788073	0.982	0.077
CN_367	388253941	8685628	27051311	0.978	0.070
CN_368	346977698	6626362	22446534	0.981	0.065
CN_369	370930901	7072350	25134060	0.981	0.068
CN_370	391878618	7318375	27768884	0.981	0.071
CN_371	362225994	5862195	26214729	0.984	0.072
CN_372	405823651	6935188	31821710	0.983	0.078
CN_373	341645436	5207599	23640246	0.985	0.069
CN_374	353067110	6525649	22428045	0.982	0.064
CN_375	377631991	7192674	30762275	0.981	0.081
CN_376	328984562	5395204	22104722	0.984	0.067
CN_377	361091411	6371772	25269465	0.982	0.070
CN_378	362071483	6401633	24962842	0.982	0.069
CN_379	322293040	4944870	20133696	0.985	0.062
CN_380	362641497	7130366	28213256	0.980	0.078
CN_381	334609344	6184478	22826492	0.982	0.068
CN_382	329987490	6716639	21474510	0.980	0.065
CN_383	345823758	5823546	22863243	0.983	0.066
CN_384	323453064	5581054	22456884	0.983	0.069
CN_385	324010714	5454880	22613230	0.983	0.070
CN_386	354423643	8004722	24602174	0.977	0.069

CN_387	334915342	5786077	22458528	0.983	0.067
CN_388	355333933	6804135	25019160	0.981	0.070
CN_389	371753150	7147371	27657202	0.981	0.074
CN_390	378371230	6025136	28162253	0.984	0.074
CN_391	335718151	5182966	22652613	0.985	0.067
CN_392	329442261	6908373	24234649	0.979	0.074
CN_393	379544127	6437382	26499538	0.983	0.070
CN_394	392448508	6315714	38670180	0.984	0.099
CN_395	362640330	7652373	22860491	0.979	0.063
CN_396	394567748	5477720	33159023	0.986	0.084
CN_397	474090035	7880980	43891142	0.983	0.093
CN_398	385769204	8000710	31271656	0.979	0.081
CN_399	376710426	7446484	25857920	0.980	0.069
CN_400	394589787	7544687	31811003	0.981	0.081
CN_401	390079528	6351786	28839518	0.984	0.074
CN_402	359595204	7371002	26595875	0.980	0.074
CN_403	350940084	7341962	23971933	0.979	0.068
CN_404	349390196	9300884	24034833	0.973	0.069
CN_405	364519232	6140064	25669818	0.983	0.070
CN_406	374006149	6667560	28565956	0.982	0.076
CN_407	335849420	7362445	23310819	0.978	0.069
CN_408	398253870	7759072	39541664	0.981	0.099
CN_409	336138357	6685731	22132586	0.980	0.066
CN_410	334998638	6671756	23297382	0.980	0.070

CN_411	357093957	7514592	26814083	0.979	0.075
CN_412	374701516	5938191	29335941	0.984	0.078
CN_413	326526860	4843810	22499040	0.985	0.069
CN_414	339436963	6176335	23784915	0.982	0.070
CN_415	448253027	8398958	35656512	0.981	0.080
CN_416	466918809	9585170	39654691	0.979	0.085
CN_417	415656228	9536592	35194929	0.977	0.085
CN_418	447679878	8267785	36946775	0.982	0.083
CN_419	459219250	8791449	44682573	0.981	0.097
CN_420	456749757	8986865	39424211	0.980	0.086
CN_421	452713484	9419229	37341535	0.979	0.082
CN_422	448285178	8126071	39536918	0.982	0.088
CN_423	451532258	9680130	38978893	0.979	0.086
CN_424	426194732	9408558	38221138	0.978	0.090
CN_425	433836407	8949530	33620078	0.979	0.077
CN_426	478420077	10842975	42623071	0.977	0.089
CN_427	459620713	6075972	44095738	0.987	0.096
CN_428	356724395	6417978	29495227	0.982	0.083
CN_429	351177182	5604384	27223453	0.984	0.078
CN_430	334562411	6295825	26640604	0.981	0.080
CN_431	333201336	5466298	23993342	0.984	0.072
CN_432	330074424	6499248	31608239	0.980	0.096
CN_433	353203416	5989890	26229012	0.983	0.074
CN_434	337670511	4709321	29478020	0.986	0.087

CN_435	331686167	5567505	24047490	0.983	0.073
CN_436	328503860	5952013	27202559	0.982	0.083
CN_437	332217692	7706468	23433978	0.977	0.071
CN_438	343087234	6365706	28904285	0.981	0.084
CN_439	349585656	5743986	27478977	0.984	0.079
CN_440	387156859	7251673	33324052	0.981	0.086
CN_441	393681908	9212103	32163090	0.977	0.082
CN_442	404610210	10147795	32651906	0.975	0.081
CN_443	400583421	7829425	34234029	0.980	0.085
CN_444	367407317	7576765	31012997	0.979	0.084
CN_445	406231141	7973782	34244857	0.980	0.084
CN_446	344729553	5632118	33161206	0.984	0.096
CN_447	429810742	6475537	36845933	0.985	0.086
CN_448	417631764	8319301	37987297	0.980	0.091
CN_449	384228623	7683189	31406599	0.980	0.082
CN_450	522946638	11883669	44682706	0.977	0.085
CN_451	501272111	10120206	39431587	0.980	0.079
CN_452	517478049	9199450	44345498	0.982	0.086
CN_453	495099165	8439352	35993375	0.983	0.073
CN_454	522342437	8485780	45483619	0.984	0.087
CN_455	515552457	7448908	40111450	0.986	0.078
CN_456	525643027	10956819	42788247	0.979	0.081
CN_457	466328787	9196190	32721582	0.980	0.070
CN_458	483340596	9101197	38914411	0.981	0.081

CN_459	474339533	8909431	32218561	0.981	0.068
CN_460	468774377	7863149	33042085	0.983	0.070
CN_461	473105855	8645363	33279228	0.982	0.070
CN_462	456366860	7902128	38008187	0.983	0.083
CN_463	483724440	8216259	34410940	0.983	0.071
CN_464	469062229	10328979	33213242	0.978	0.071
CN_465	465044941	8506168	34664111	0.982	0.075
CN_466	490174105	6442112	36509231	0.987	0.074
CN_467	335399723	6580565	22935844	0.980	0.068
CN_468	380442800	6915874	30271996	0.982	0.080
CN_469	344788925	6900643	22563142	0.980	0.065
CN_470	320726172	5215240	20167999	0.984	0.063
CN_471	379403085	5299330	31042202	0.986	0.082
CN_472	333092160	6474397	22151400	0.981	0.067
CN_473	380890668	7493412	27819573	0.980	0.073
CN_474	334038972	5886479	23780190	0.982	0.071
CN_475	356233410	6136256	23396161	0.983	0.066
CN_476	347204978	7708161	24440221	0.978	0.070
CN_477	364759849	8386602	24496760	0.977	0.067
CN_478	385297114	8422045	29701246	0.978	0.077
CN_479	350130277	6127276	22737506	0.983	0.065
CN_480	345521484	6178206	23277988	0.982	0.067
CN_481	374218466	6798837	25780577	0.982	0.069
CN_482	382359168	5358319	29417355	0.986	0.077

CN_483	361727566	5838232	25184793	0.984	0.070
CN_484	372635546	6263336	24888414	0.983	0.067
CN_485	389001442	10831233	28757852	0.972	0.074
CN_486	356391643	5724853	24120565	0.984	0.068
CN_487	376717964	7400922	34961862	0.980	0.093
CN_488	370589833	5527232	24639218	0.985	0.066
CN_489	374396739	5882578	26713616	0.984	0.071
CN_490	406488242	5433015	30516822	0.987	0.075
CN_491	378900859	5526679	29221669	0.985	0.077
CN_492	376395783	6406969	28223443	0.983	0.075
CN_493	386506416	7149870	26583679	0.982	0.069
CN_494	351098516	5626912	21674203	0.984	0.062
CN_495	391627669	7382107	33755736	0.981	0.086
CN_496	360619994	6821017	23769106	0.981	0.066
CN_497	365594469	5657449	24972125	0.985	0.068
CN_498	341036293	6495197	23240414	0.981	0.068
CN_499	357645223	5566969	25056449	0.984	0.070
CN_500	382701752	6049003	28407402	0.984	0.074
CN_501	345463627	7833893	23070599	0.977	0.067
CN_502	341567293	5122772	22453190	0.985	0.066
CN_503	378574982	5849290	29156712	0.985	0.077
CN_504	359377317	6380665	23919696	0.982	0.067
CN_505	344292144	7473045	21666421	0.978	0.063
CN_506	366231688	6216277	29375925	0.983	0.080

CN_507	331867322	5903000	21030537	0.982	0.063
CN_508	318406336	5588325	18653267	0.982	0.059
CN_509	353607836	6200235	26459963	0.982	0.075
CN_510	352421719	5995811	19832198	0.983	0.056
CN_511	342348881	6775275	18790317	0.980	0.055
CN_512	352749341	5618472	19241772	0.984	0.055
CN_513	356416764	5008478	20858522	0.986	0.059
CN_514	357021970	6277762	20018813	0.982	0.056
CN_515	364954078	6527234	20565494	0.982	0.056
CN_516	339937022	5449743	18537625	0.984	0.055
CN_517	353183493	4611157	20454666	0.987	0.058
CN_518	373217672	5869121	24364981	0.984	0.065
CN_519	387608904	4866792	24040529	0.987	0.062
CN_520	357539131	5271713	19524542	0.985	0.055
CN_521	377993610	5321760	23395203	0.986	0.062
CN_522	358257209	6037932	21130232	0.983	0.059
CN_523	356165599	6187457	20144797	0.983	0.057
CN_524	373689531	7395322	22228226	0.980	0.059
CN_525	381485826	6270957	22032030	0.984	0.058
CN_526	355834909	5224212	28373108	0.985	0.080
CN_527	369376976	6561057	19664099	0.982	0.053
CN_528	398242370	4711769	26552272	0.988	0.067
CN_529	387462728	7343880	23576705	0.981	0.061
CN_530	355337356	5682391	24035581	0.984	0.068

CN_531	350928934	6432204	20662628	0.982	0.059
CN_532	345348802	5195199	23267013	0.985	0.067
CN_533	348851487	4770469	21282137	0.986	0.061
CN_534	376877745	5466352	22823233	0.985	0.061
CN_535	362581873	6618085	20068819	0.982	0.055
CN_536	367648285	7821010	19508883	0.979	0.053
CN_537	382048006	6774116	22990108	0.982	0.060
CN_538	349996646	6263924	19749484	0.982	0.056
CN_539	361952957	5990842	31144897	0.983	0.086
CN_540	343309938	4693191	17751604	0.986	0.052
CN_541	367604258	8101750	21038326	0.978	0.057
CN_542	367594915	7459378	22064948	0.980	0.060
CN_543	384102948	6249405	25276111	0.984	0.066
CN_544	356761644	6297489	27987296	0.982	0.078
CN_545	361637681	7270559	30783104	0.980	0.085
CN_546	431958226	6587711	40113930	0.985	0.093
CN_547	348373978	6265293	29752153	0.982	0.085
CN_548	439251851	11523293	38031668	0.974	0.087
CN_549	348603477	5380327	22679976	0.985	0.065
CN_550	387910844	6880208	25836041	0.982	0.067
CN_551	340710569	5306146	21111934	0.984	0.062
CN_552	344764426	6006600	20051893	0.983	0.058
CN_553	340614428	6880131	21194198	0.980	0.062
CN_554	346006287	6396292	20264871	0.982	0.059

CN_555	376655596	7295112	26939965	0.981	0.072
CN_556	370299287	5779370	22794826	0.984	0.062
CN_557	345821800	5599099	20833477	0.984	0.060
CN_558	355993097	6913429	21502866	0.981	0.060
CN_559	361240215	6658566	22393809	0.982	0.062
CN_560	348384549	6473652	21540029	0.981	0.062
CN_561	375987959	7479722	23567738	0.980	0.063
CN_562	358616185	6309207	21414199	0.982	0.060
CN_563	392078040	6755173	26215867	0.983	0.067
CN_564	389995664	6673154	26186166	0.983	0.067
CN_565	367463741	5932679	22772373	0.984	0.062
CN_566	352714269	6778687	22795373	0.981	0.065
CN_567	380184210	6440327	23754310	0.983	0.062
CN_568	388677298	6546151	26802231	0.983	0.069
CN_569	392108186	5844961	25158288	0.985	0.064
CN_570	369479132	6899609	22671045	0.981	0.061
CN_571	399881838	5335791	29287145	0.987	0.073
CN_572	380225227	6002889	24825832	0.984	0.065
CN_573	398429056	7163303	25062168	0.982	0.063
CN_574	366063079	5364846	26894478	0.985	0.073
CN_575	397111920	6610147	26448804	0.983	0.067
CN_576	384209630	6019764	27479378	0.984	0.072
CN_577	373538562	7718930	23499103	0.979	0.063
CN_578	376412798	9013073	21781163	0.976	0.058

CN_579	368106683	5472720	24555570	0.985	0.067
CN_580	396832713	6400324	27215958	0.984	0.069
CN_581	364577402	7030009	22845631	0.981	0.063
CN_582	367820559	5678072	33522768	0.985	0.091
CN_583	364337029	7927207	23262036	0.978	0.064
CN_584	383241381	7131632	26104391	0.981	0.068
CN_585	361888398	6141000	24676867	0.983	0.068
CN_586	347150494	6019656	21317533	0.983	0.061
CN_587	393395150	5968470	28146566	0.985	0.072
CN_588	374063820	6346031	21154281	0.983	0.057
CN_589	390053808	7422348	27151259	0.981	0.070
CN_590	371639209	4932923	23261019	0.987	0.063
CN_591	366014881	6846220	24057604	0.981	0.066
CN_592	373331680	7530542	23343876	0.980	0.063
CN_593	383012721	5830141	26054428	0.985	0.068
CN_594	379529026	7451995	22711392	0.980	0.060
CN_595	374048605	6267355	23857589	0.983	0.064
CN_596	343631444	4916765	21050497	0.986	0.061
CN_597	369737284	7014724	27206206	0.981	0.074
CN_598	371562321	7637053	22713788	0.979	0.061
CN_599	352027256	7381808	21602774	0.979	0.061
CN_600	373635772	7851822	24271488	0.979	0.065
CN_601	395970291	4308735	26586149	0.989	0.067
CN_602	340448167	5267163	21203032	0.985	0.062

CN_603	374019497	6155231	27023931	0.984	0.072
CN_604	346621931	7649099	21536422	0.978	0.062
CN_605	332126148	4914162	21170542	0.985	0.064
CN_606	366481460	5452823	26230870	0.985	0.072
CN_607	327716800	5534258	20680290	0.983	0.063
CN_608	366789400	7300894	25142915	0.980	0.069
CN_609	340285211	5648866	22059353	0.983	0.065
CN_610	334292292	4727846	21518135	0.986	0.064
CN_611	330596726	6416756	21106954	0.981	0.064
CN_612	359611239	7857063	22537814	0.978	0.063
CN_613	371159320	7436309	26171170	0.980	0.071
CN_614	339235875	6198687	20707899	0.982	0.061
CN_615	332261648	6408960	21517654	0.981	0.065
CN_616	348525702	6528159	22463240	0.981	0.064
CN_617	366872319	6032468	25399158	0.984	0.069
CN_618	344945844	5359663	23261476	0.984	0.067
CN_619	339208182	5474239	21227525	0.984	0.063
CN_620	382324530	10628305	25185730	0.972	0.066
CN_621	342285585	5378467	22183431	0.984	0.065
CN_622	380875074	6926660	30109125	0.982	0.079
CN_623	357669468	7524628	23110930	0.979	0.065
CN_624	366289901	8010190	23501182	0.978	0.064
CN_625	369830018	6141800	24953385	0.983	0.067
CN_626	360004036	6999629	25847772	0.981	0.072

CN_627	358226385	5854581	25975083	0.984	0.073
CN_628	370062944	8102452	22223610	0.978	0.060
CN_629	334317254	5294797	20452963	0.984	0.061
CN_630	374455943	7062016	27930478	0.981	0.075
CN_631	345222025	6106800	21791629	0.982	0.063
CN_632	359413937	7081684	23174860	0.980	0.064
CN_633	349091365	7146534	22644541	0.980	0.065
CN_634	356992302	6675675	24150264	0.981	0.068
CN_635	376414726	5554838	26723149	0.985	0.071
CN_636	347770112	6538817	22477522	0.981	0.065
CN_637	359899992	5500478	24479423	0.985	0.068
CN_638	379833821	5860247	26956571	0.985	0.071
CN_639	330994642	4565429	22325803	0.986	0.067
CN_640	360862975	5686796	25002220	0.984	0.069
CN_641	342424981	7046427	22312102	0.979	0.065
CN_642	342380502	7830050	19818371	0.977	0.058
CN_643	375665730	7148526	27796431	0.981	0.074
CN_644	337969487	5138699	21480465	0.985	0.064
CN_645	364105336	6096229	26589724	0.983	0.073
CN_646	367149548	6617708	23473697	0.982	0.064
CN_647	368142251	7835394	22756974	0.979	0.062
CN_648	331444663	5231002	22035635	0.984	0.066
CN_649	334618589	6253129	21500288	0.981	0.064
CN_650	361113647	8019031	23364918	0.978	0.065

CN_651	356345077	6422088	22286927	0.982	0.063
CN_652	350130663	6052902	22771316	0.983	0.065
CN_653	366529871	7626481	25264833	0.979	0.069
CN_654	361221707	5512130	23989126	0.985	0.066
CN_655	366194043	5743418	23986197	0.984	0.066
CN_656	373124338	5505501	26865415	0.985	0.072
CN_657	299835000	3344564	23650781	0.989	0.079
CN_658	351325364	2513050	43718312	0.993	0.124
CN_659	298991831	2772481	24467612	0.991	0.082
CN_660	301389552	3180085	24844677	0.989	0.082
CN_661	304681550	3408819	27830003	0.989	0.091
CN_662	300771599	3087036	23991263	0.990	0.080
CN_663	311949130	3730691	29004078	0.988	0.093
CN_664	308127236	4194677	25646062	0.986	0.083
CN_665	306261578	3415605	25072662	0.989	0.082
CN_666	401549133	3643255	36280444	0.991	0.090
CN_667	312760549	3045961	27432765	0.990	0.088
CN_668	312901410	3353807	28907594	0.989	0.092
CN_669	302770476	3160814	24281011	0.990	0.080
CN_670	388340966	3524927	38325244	0.991	0.099
CN_671	344362334	3061577	34431696	0.991	0.100
CN_672	397709847	4019205	36969000	0.990	0.093
CN_673	394715719	4505144	36175290	0.989	0.092
CN_674	353428466	3191526	31413545	0.991	0.089

CN_675	389594029	4981266	25611147	0.987	0.066
CN_676	371984436	6790381	40727118	0.982	0.109
CN_677	374723915	7321420	36981951	0.980	0.099
CN_678	382103786	6351800	26583450	0.983	0.070
CN_679	360056998	8683386	18778516	0.976	0.052
CN_680	315144243	3603580	28268495	0.989	0.090
CN_681	392540415	6846427	38038237	0.983	0.097
CN_682	409764236	4037606	39232357	0.990	0.096
CN_683	416170269	2947402	38999982	0.993	0.094
CN_684	355864540	9543686	34687804	0.973	0.097
CN_685	332624867	3785607	33219869	0.989	0.100
CN_686	346645629	6859077	22429883	0.980	0.065
CN_687	371777838	6238361	26284586	0.983	0.071
CN_688	333521202	6039395	22015532	0.982	0.066
CN_689	325521040	3495264	32821280	0.989	0.101
CN_690	342271552	3910786	28808072	0.989	0.084
CN_691	319337384	2901252	25555090	0.991	0.080
CN_692	336521461	5689039	19465663	0.983	0.058
CN_693	371601348	6789221	34765946	0.982	0.094
CN_694	360392680	7367416	22145368	0.980	0.061
CN_695	338194930	5652088	21614123	0.983	0.064
CN_696	412067683	3725289	38323631	0.991	0.093
CN_697	392173912	6647967	23304651	0.983	0.059
CN_698	341378784	3186598	35159437	0.991	0.103

CN_699	385313558	7309859	39506192	0.981	0.103
CN_700	354368480	4665164	20288886	0.987	0.057
CN_701	343758430	7608923	19375267	0.978	0.056
CN_702	371045702	7138321	28613034	0.981	0.077
CN_703	385961784	6488635	42214936	0.983	0.109
CN_704	300161210	3076877	25181607	0.990	0.084
CN_705	358397296	5227142	21690543	0.985	0.061
CN_706	359882723	3466524	30774281	0.990	0.086
CN_707	377888955	7066046	23116476	0.981	0.061
CN_708	324779405	4503662	17419275	0.986	0.054
CN_709	394805009	8006635	45189254	0.980	0.114
CN_710	325553150	3476633	26903421	0.989	0.083
CN_711	393841858	5914111	32891264	0.985	0.084
CN_712	361204089	5506737	23638645	0.985	0.065
CN_713	351286971	6057535	19240391	0.983	0.055
CN_714	367906269	7790675	38686550	0.979	0.105
CN_715	374736917	5894289	43239196	0.984	0.115
CN_716	335076894	6563142	30423082	0.980	0.091
CN_717	300381579	3552210	23290287	0.988	0.078
CN_718	358963528	6912771	34513477	0.981	0.096
CN_719	360592096	6140877	21236353	0.983	0.059
CN_720	373846420	6872533	43099989	0.982	0.115
CN_721	454360085	9766381	37241753	0.979	0.082
CN_722	351912903	5913919	23836397	0.983	0.068

CN_723	368087781	6682974	21979109	0.982	0.060
CN_724	364590503	7110736	21705864	0.980	0.060
CN_725	329432020	7057543	18758313	0.979	0.057
CN_726	362995238	9121399	32285687	0.975	0.089
CN_727	297454431	2968422	23629962	0.990	0.079
CN_728	316406405	5084801	16816351	0.984	0.053
CN_729	370866470	5578422	20737966	0.985	0.056
CN_730	324705285	3520551	27259833	0.989	0.084
CN_731	332143124	6306973	18360226	0.981	0.055
CN_732	341589578	3821648	29159117	0.989	0.085
CN_733	376313757	6686649	23130681	0.982	0.061
CN_734	382652577	7049214	28532079	0.982	0.075
CN_735	359822310	6462039	21132398	0.982	0.059
CN_736	358230419	7264046	21270517	0.980	0.059
CN_737	331381575	6583569	18095060	0.980	0.055
CN_738	317661696	2892111	26919943	0.991	0.085
CN_739	359970342	7768895	19951373	0.978	0.055
CN_740	307897083	3546524	24953719	0.988	0.081
CN_741	356312088	5349189	22316157	0.985	0.063
CN_742	337977846	5549398	21338762	0.984	0.063
CN_743	383103070	11133070	22673130	0.971	0.059
CN_744	338892942	6578579	22769356	0.981	0.067
CN_745	372993886	4307702	38816651	0.988	0.104
CN_746	368660006	6006111	32626197	0.984	0.088

CN_747	350037066	8254171	21715913	0.976	0.062
CN_748	404289458	4568881	38932153	0.989	0.096
CN_749	308578069	3107848	24176052	0.990	0.078
CN_750	299845744	3200067	25078258	0.989	0.084
CN_751	365018308	4872832	23105150	0.987	0.063
CN_752	301685381	2645190	24442840	0.991	0.081
CN_753	351578626	6960283	18120569	0.980	0.052
CN_754	380677815	2810191	36050524	0.993	0.095
CN_755	355258309	6549935	20158136	0.982	0.057
CN_756	392907423	3445073	37203182	0.991	0.095
CN_757	363347780	8071125	36630897	0.978	0.101
CN_758	327436290	6384953	17801376	0.981	0.054
CN_759	337960986	7288022	31045179	0.978	0.092
CN_760	394057895	8652870	34326340	0.978	0.087
CN_761	335407146	6620700	18489068	0.980	0.055
CN_762	361884353	6427453	20439025	0.982	0.056
CN_763	352121353	10036195	33647680	0.971	0.096
CN_764	359548532	6843588	35828582	0.981	0.100
CN_765	377732377	7387061	36011740	0.980	0.095
CN_766	397646358	6919621	41317244	0.983	0.104
CN_767	378022588	6548652	36262075	0.983	0.096
CN_768	387462975	7034551	25237799	0.982	0.065
CN_769	340601372	5965491	18693289	0.982	0.055
CN_770	363494369	5619202	23405542	0.985	0.064

CN_771	350743356	6002509	18937552	0.983	0.054
CN_772	381328080	6027327	29684093	0.984	0.078
CN_773	357779480	7890891	20427807	0.978	0.057
CN_774	351919360	6279168	18684587	0.982	0.053
CN_775	386949638	5106488	25875782	0.987	0.067
CN_776	364754768	6212868	20054833	0.983	0.055
CN_777	415706239	7537785	31683346	0.982	0.076
CN_778	369853934	6876528	29056453	0.981	0.079
CN_779	375718832	7947898	22958359	0.979	0.061
CN_780	390179647	7330257	24713788	0.981	0.063
CN_781	390482092	8114100	30000195	0.979	0.077
CN_782	379711460	6971330	21729237	0.982	0.057
CN_783	389240802	6967112	26017648	0.982	0.067
CN_784	386097150	6622437	22775824	0.983	0.059
CN_785	419874954	4943183	29637301	0.988	0.071
CN_786	370332353	6465277	22379090	0.983	0.060
CN_787	378252367	9346632	19813783	0.975	0.052
CN_788	390510700	7498533	31157122	0.981	0.080
CN_789	335876089	6658285	16913969	0.980	0.050
CN_790	345139751	7018603	17668425	0.980	0.051
CN_791	322986005	4755102	18224426	0.985	0.056
CN_792	339823425	7430985	19050814	0.978	0.056
CN_793	376147772	7178781	23870675	0.981	0.063
CN_794	340819446	6423709	25825352	0.981	0.076

CN_795	345494526	2544908	35046749	0.993	0.101
CN_796	359671122	6403682	18976502	0.982	0.053
CN_797	328958740	5487281	18546846	0.983	0.056
CN_798	390765262	5645652	29443535	0.986	0.075

AD, Alzheimer disease; CN, cognitively normal control
