# Supplementary material for: Whole-genome sequencing reveals novel ethnicity-specific rare variants associated with Alzheimer’s disease
Source: Mol Psychiatry. 2022 Mar 10;27(5):2554–62. doi: 10.1038/s41380-022-01483-0 (PMC9135624; doi:10.1038/s41380-022-01483-0)
Supplement: Supplementary file 5 — Table S2 [file 41380_2022_1483_MOESM5_ESM.pdf]

Table S2. A total of 300 candidate genes showing an association with AD at P_{BH} < 0.01

gene symbol	gene ID	SetSize	P	P_{BH}	P_{bon}
MLKL	197259	6	2.09E-06	0.010	**0.010**
DNAH8	1769	13	1.87E-05	0.030	0.092
TTN	7273	88	2.38E-05	0.030	0.117
TRPM1	4308	6	2.75E-05	0.030	0.135
SH3RF2	153769	8	3.10E-05	0.030	0.152
OSBPL5	114879	2	4.71E-05	0.039	0.232
LRBA	987	8	6.22E-05	0.039	0.306
GLCCI1	113263	2	6.71E-05	0.039	0.330
FAM13A	10144	3	7.23E-05	0.039	0.355
RASGRF2	5924	3	0.0001	0.049	0.494
PDS5B	23047	2	0.0001	0.060	0.666
ZYG11A	440590	4	0.0001	0.060	0.737
PRSS57	400668	3	0.0002	0.060	0.799
SBF2	81846	4	0.0002	0.060	0.841
PPFIA4	8497	5	0.0002	0.060	0.972
SAMD9L	219285	5	0.0002	0.060	0.972
FARP1	10160	4	0.0002	0.060	1
SHE	126669	2	0.0002	0.060	1
ARAP3	64411	3	0.0002	0.060	1
PLEKHG3	26030	4	0.0002	0.060	1
DNAH11	8701	26	0.0003	0.060	1
OR51G1	79324	6	0.0003	0.060	1

PCNT	5116	14	0.0003	0.060	1
PLEC	5339	27	0.0003	0.060	1
C1orf167	284498	8	0.0003	0.061	1
LGR5	8549	5	0.0003	0.061	1
GNPTAB	79158	6	0.0004	0.061	1
NEDD4	4734	6	0.0004	0.061	1
RHBDF2	79651	4	0.0004	0.061	1
CRB1	23418	2	0.0004	0.061	1
NCOR2	9612	9	0.0004	0.061	1
DENND3	22898	4	0.0004	0.061	1
H6PD	9563	9	0.0004	0.061	1
MORC2	22880	2	0.0004	0.061	1
PGC	5225	3	0.0005	0.065	1
BTAF1	9044	4	0.0005	0.069	1
ZNF883	169834	4	0.0005	0.069	1
TLR1	7096	5	0.0005	0.069	1
GLIS2	84662	2	0.0006	0.071	1
PCDHA12	56137	3	0.0006	0.079	1
CEP70	80321	2	0.0007	0.080	1
PF4	5196	2	0.0007	0.082	1
ANKLE1	126549	2	0.0007	0.083	1
SLC37A1	54020	3	0.0008	0.083	1
PPM1E	22843	3	0.0008	0.083	1
EXO1	9156	2	0.0008	0.083	1

ESR2	2100	3	0.0009	0.083	1
ZNF221	7638	2	0.0009	0.083	1
TAS2R40	259286	4	0.0009	0.083	1
TDRD12	91646	6	0.0009	0.083	1
PIK3C2G	5288	5	0.0009	0.083	1
ZNF613	79898	3	0.0009	0.083	1
OR4X2	119764	2	0.0009	0.084	1
PTPRU	10076	5	0.0010	0.084	1
DYSF	8291	9	0.0010	0.084	1
ZNF714	148206	4	0.0010	0.084	1
SPATA21	374955	4	0.0010	0.084	1
RNF213	57674	10	0.0010	0.084	1
EYA1	2138	5	0.0011	0.086	1
CORIN	10699	5	0.0011	0.086	1
ABCA13	154664	12	0.0011	0.086	1
PTPRN	5798	3	0.0011	0.088	1
DMD	1756	13	0.0012	0.088	1
KRT9	3857	2	0.0012	0.088	1
DST	667	12	0.0012	0.088	1
PDIA5	10954	3	0.0013	0.099	1
LMTK2	22853	4	0.0014	0.099	1
PAPPA	5069	8	0.0014	0.102	1
CHST3	9469	2	0.0015	0.108	1
TBCCD1	55171	2	0.0016	0.112	1

MYH11	4629	4	0.0016	0.112	1
ZNF479	90827	3	0.0017	0.112	1
KCNE1	3753	2	0.0017	0.112	1
NVL	4931	4	0.0017	0.112	1
RIMS2	9699	4	0.0017	0.114	1
CR2	1380	3	0.0018	0.115	1
GREB1	9687	7	0.0019	0.115	1
ADAMTSL1	92949	13	0.0019	0.115	1
OAS2	4939	3	0.0019	0.115	1
DYNC1H1	1778	4	0.0019	0.115	1
RNF222	643904	2	0.0020	0.115	1
LY9	4063	4	0.0020	0.115	1
PCLO	27445	7	0.0020	0.115	1
ALDH16A1	126133	2	0.0020	0.115	1
AFP	174	3	0.0020	0.115	1
AKNA	80709	4	0.0020	0.115	1
TRIM59	286827	2	0.0020	0.115	1
CRTAC1	55118	5	0.0021	0.117	1
ZNF862	643641	4	0.0021	0.118	1
PDCD11	22984	8	0.0022	0.118	1
PLA2G4D	283748	6	0.0023	0.123	1
PLA2G4C	8605	3	0.0024	0.127	1
IGSF8	93185	5	0.0024	0.127	1
NCKIPSD	51517	2	0.0024	0.127	1

MYOM3	127294	7	0.0026	0.133	1
FGFBP3	143282	3	0.0026	0.133	1
FAM126A	84668	6	0.0027	0.134	1
MVP	9961	4	0.0027	0.134	1
RASAL2	9462	5	0.0027	0.134	1
FNDC1	84624	6	0.0028	0.134	1
OR2T7	81458	2	0.0028	0.134	1
ENGASE	64772	4	0.0028	0.134	1
SIGIRR	59307	2	0.0029	0.134	1
RYR2	6262	5	0.0030	0.134	1
ARHGEF18	23370	7	0.0030	0.134	1
MAP1S	55201	6	0.0030	0.134	1
USP26	83844	2	0.0031	0.134	1
OLFML2A	169611	4	0.0031	0.134	1
WNK2	65268	11	0.0031	0.134	1
PNKD	25953	3	0.0032	0.134	1
SCNN1B	6338	2	0.0032	0.134	1
CCDC168	643677	18	0.0032	0.134	1
GMCL1	64395	2	0.0032	0.134	1
TNC	3371	9	0.0033	0.134	1
CYP4F3	4051	4	0.0033	0.134	1
IPPK	64768	3	0.0033	0.134	1
PRUNE2	158471	8	0.0033	0.134	1
NCAPG2	54892	2	0.0033	0.134	1

HIST1H1C	3006	3	0.0034	0.134	1
DNAH9	1770	14	0.0034	0.134	1
ADAM28	10863	4	0.0035	0.134	1
ABCA7	10347	11	0.0035	0.134	1
MED1	5469	2	0.0035	0.134	1
CCDC57	284001	5	0.0035	0.134	1
APC	324	8	0.0035	0.134	1
JSRP1	126306	2	0.0036	0.134	1
ACTR8	93973	2	0.0036	0.134	1
SLC22A7	10864	2	0.0036	0.134	1
PKP3	11187	3	0.0036	0.134	1
FAM13B	51306	2	0.0036	0.134	1
NMUR1	10316	6	0.0036	0.134	1
GANAB	23193	5	0.0036	0.134	1
TSGA10	80705	2	0.0036	0.134	1
MADCAM1	8174	3	0.0037	0.134	1
PDCL2	132954	2	0.0037	0.134	1
BCAM	4059	3	0.0038	0.134	1
HSPG2	3339	14	0.0038	0.134	1
PLXND1	23129	5	0.0038	0.134	1
CSPG4	1464	8	0.0039	0.134	1
NOX5	79400	5	0.0039	0.134	1
PDE4DIP	9659	10	0.0039	0.134	1
SPTBN2	6712	7	0.0039	0.134	1

NAALAD2	10003	2	0.0040	0.134	1
KCNH3	23416	3	0.0040	0.134	1
ZNF230	7773	2	0.0040	0.134	1
AMPD1	270	5	0.0041	0.134	1
ZDBF2	57683	5	0.0041	0.134	1
ARHGAP40	343578	4	0.0041	0.134	1
KIAA1324L	222223	2	0.0041	0.134	1
DPY19L4	286148	4	0.0041	0.134	1
NCR3	259197	3	0.0042	0.134	1
GPR132	29933	2	0.0042	0.134	1
KIAA1614	57710	4	0.0042	0.134	1
PIEZO2	63895	5	0.0042	0.135	1
ANGPTL7	10218	4	0.0042	0.135	1
PLOD2	5352	3	0.0043	0.135	1
SLFN12L	100506736	2	0.0043	0.135	1
ELMO3	79767	5	0.0044	0.136	1
ITGB4	3691	4	0.0044	0.136	1
STAG3	10734	2	0.0044	0.136	1
LRRC37B	114659	3	0.0045	0.136	1
MYH7	4625	3	0.0045	0.136	1
NCAN	1463	5	0.0046	0.136	1
PGAP1	80055	3	0.0046	0.136	1
TNN	63923	5	0.0046	0.136	1
PLBD2	196463	2	0.0047	0.136	1

TTC34	100287898	4	0.0047	0.136	1
CROCC	9696	13	0.0047	0.136	1
SLIT1	6585	5	0.0048	0.136	1
COL22A1	169044	8	0.0048	0.136	1
ZNF544	27300	3	0.0049	0.136	1
FAM186A	121006	3	0.0049	0.136	1
COL27A1	85301	6	0.0049	0.136	1
TRIM50	135892	2	0.0049	0.136	1
TG	7038	10	0.0049	0.136	1
WDR72	256764	3	0.0049	0.136	1
UHRF1BP1L	23074	3	0.0049	0.136	1
MKRN2	23609	2	0.0049	0.136	1
VEGFB	7423	3	0.0050	0.137	1
AKAP12	9590	7	0.0050	0.137	1
SIM1	6492	2	0.0050	0.137	1
EHD4	30844	5	0.0051	0.137	1
SLC7A8	23428	3	0.0052	0.139	1
GTF2A1L	11036	2	0.0052	0.139	1
TARBP1	6894	9	0.0053	0.139	1
FSIP2	401024	19	0.0053	0.139	1
ISX	91464	2	0.0053	0.139	1
LYST	1130	5	0.0053	0.139	1
PPP1R3F	89801	2+D200	0.0054	0.139	1
DGKH	160851	5	0.0055	0.141	1

SFXN2	118980	3	0.0055	0.142	1
MYO7B	4648	7	0.0056	0.144	1
ARHGEF4	50649	8	0.0057	0.144	1
MUC5B	727897	21	0.0057	0.144	1
CLMN	79789	3	0.0058	0.144	1
ARMC7	79637	3	0.0058	0.144	1
IQCE	23288	5	0.0058	0.144	1
SLC12A3	6559	2	0.0058	0.144	1
HLA-A	3105	6	0.0059	0.145	1
ABI3BP	25890	7	0.0060	0.146	1
DNAH1	25981	15	0.0060	0.146	1
AP4B1	10717	4	0.0060	0.146	1
ZDHHC5	25921	3	0.0061	0.147	1
GLRX2	51022	3	0.0061	0.147	1
RPS6KB2	6199	4	0.0061	0.147	1
SERPINB7	8710	3	0.0062	0.147	1
AGR3	155465	4	0.0062	0.147	1
SZT2	23334	4	0.0065	0.152	1
EFCAB5	374786	6	0.0065	0.152	1
NBEAL2	23218	7	0.0066	0.153	1
STARD13	90627	2	0.0066	0.153	1
RBP3	5949	6	0.0067	0.156	1
SEZ6	124925	3	0.0068	0.156	1
CNTRL	11064	6	0.0068	0.157	1

CELSR1	9620	9	0.0069	0.158	1
PTPN3	5774	2	0.0069	0.158	1
LAMA5	3911	12	0.0070	0.159	1
COL6A5	256076	6	0.0071	0.159	1
MLLT10	8028	3	0.0073	0.159	1
FLG	2312	30	0.0073	0.159	1
SVEP1	79987	7	0.0073	0.159	1
ENDOV	284131	2	0.0074	0.159	1
ZNF16	7564	2	0.0074	0.159	1
ZC3H12A	80149	3	0.0075	0.159	1
RAD52	5893	3	0.0075	0.159	1
MGA	23269	9	0.0075	0.159	1
TLX1	3195	3	0.0075	0.159	1
BMS1	9790	4	0.0075	0.159	1
ZNF747	65988	3	0.0075	0.159	1
STXBP2	6813	2	0.0076	0.159	1
XPC	7508	4	0.0076	0.159	1
CNTROB	116840	4	0.0076	0.159	1
ARMC3	219681	4	0.0077	0.159	1
QSER1	79832	3	0.0078	0.159	1
MMP15	4324	4	0.0078	0.159	1
TSEN54	283989	3	0.0078	0.159	1
PRDM4	11108	4	0.0079	0.159	1
CATSPER1	117144	7	0.0079	0.159	1

AK7	122481	5	0.0081	0.159	1
TRDN	10345	2	0.0082	0.159	1
DHRS1	115817	3	0.0082	0.159	1
AGBL4	84871	2	0.0083	0.159	1
KLC3	147700	5	0.0083	0.159	1
ADH4	127	4	0.0084	0.159	1
RNF212	285498	5	0.0085	0.159	1
OAS3	4940	5	0.0085	0.159	1
THEMIS	387357	2	0.0086	0.159	1
AKNAD1	254268	5	0.0087	0.159	1
CC2D2B	387707	9	0.0087	0.159	1
SYNE2	23224	17	0.0087	0.159	1
SUMF2	25870	2	0.0087	0.159	1
ARHGEF35	445328	2	0.0087	0.159	1
DDX4	54514	3	0.0088	0.159	1
STK36	27148	5	0.0088	0.159	1
DAPK2	23604	4	0.0089	0.159	1
FAT3	120114	10	0.0089	0.159	1
BNC2	54796	4	0.0089	0.159	1
ANKRD35	148741	3	0.0090	0.159	1
NOTCH4	4855	4	0.0090	0.159	1
PRDM2	7799	6	0.0091	0.159	1
ARHGAP23	57636	5	0.0091	0.159	1
PUS7L	83448	3	0.0091	0.159	1

LLGL1	3996	3	0.0092	0.159	1
MYEOV	26579	3	0.0092	0.159	1
CHD6	84181	5	0.0092	0.159	1
NPBWR2	2832	2	0.0092	0.159	1
IARS2	55699	3	0.0093	0.159	1
KRTAP10-1	386677	3	0.0093	0.159	1
SNX29	92017	2	0.0093	0.159	1
SRPK1	6732	2	0.0093	0.159	1
OR51E1	143503	3	0.0094	0.159	1
IQSEC1	9922	2	0.0094	0.159	1
ATG4C	84938	2	0.0094	0.159	1
SGSM3	27352	2	0.0094	0.159	1
ZNF594	84622	6	0.0095	0.159	1
DHDH	27294	2	0.0095	0.159	1
CIAPIN1	57019	2	0.0095	0.159	1
COMMD1	150684	2	0.0096	0.159	1
SLC22A6	9356	2	0.0096	0.159	1
PAQR6	79957	2	0.0096	0.159	1
BACH1	571	2	0.0096	0.159	1
OR10Z1	128368	2	0.0096	0.159	1
KIFC3	3801	2	0.0096	0.159	1
PLIN4	729359	7	0.0096	0.159	1
CITED2	10370	2	0.0096	0.159	1
TRAFD1	10906	2	0.0096	0.159	1

CNGA4	1262	2	0.0096	0.159	1
DNAJC5G	285126	3	0.0097	0.159	1
HMCN1	83872	13	0.0097	0.159	1
THEG	51298	2	0.0097	0.159	1
UMOD	7369	2	0.0097	0.159	1
LANCL1	10314	2	0.0097	0.159	1
EGFLAM	133584	2	0.0097	0.159	1
ADAMTS4	9507	4	0.0098	0.159	1
KRTAP10-11	386678	2	0.0099	0.159	1
ZNF721	170960	3	0.0099	0.159	1
CLIC6	54102	2	0.0099	0.159	1
ASPG	374569	2	0.0099	0.159	1
CSRNP1	64651	2	0.0100	0.159	1
CPNE7	27132	2	0.0100	0.159	1

P: genome-wide gene-based burden testing on coding rare variants.

P_{BH}: Benjamini-Hochberg (BH) corrected P.

P_{bon}: Bonferroni-corrected P.
